# Cabergoline: a review of its use in the inhibition of lactation for women living with HIV

**DOI:** 10.1002/jia2.25322

**Published:** 2019-06-11

**Authors:** Karen J Tulloch, Philippe Dodin, Fannie Tremblay‐Racine, Chelsea Elwood, Deborah Money, Isabelle Boucoiran

**Affiliations:** ^1^ Department of Pharmacy BC Women's Hospital and Health Centre Vancouver BC Canada; ^2^ Women's Health Research Institute Vancouver BC Canada; ^3^ CHU Sainte‐Justine Montreal QC Canada; ^4^ Department of Obstetrics and Gynaecology University of British Columbia Vancouver BC Canada; ^5^ Department of Obstetrics and Gynecology CHU Sainte‐Justine Université de Montréal Montreal QC Canada

**Keywords:** cabergoline, lactation inhibition, lactation suppression, post‐partum, HIV

## Abstract

**Introduction:**

In developed countries, breastfeeding is not recommended for women living with human immunodeficiency virus (WLWH). However, lactation symptoms can be distressing for women who choose not to breastfeed. There is currently no universal guideline on the most appropriate options for prevention or reduction of lactation symptoms amongst WLWH. This review describes the evidence base for using cabergoline, a dopaminergic agonist, for the post‐partum inhibition of lactation for WLWH.

**Methods:**

A scoping review of post‐partum pharmaceutical lactation inhibition specific for WLWH was conducted using searches in PubMed, Medline Ovid, EBM Reviews Ovid, Embase, Web of Science and Scopus until 2019. A narrative review of cabergoline pharmacologic properties, therapeutic efficacy, tolerability data and drug interaction data relevant to lactation inhibition was then conducted.

**Results and discussion:**

Among 1366 articles, the scoping review identified 13 relevant publications. Eight guidelines providing guidance regarding lactation inhibition for WLWH and two surveys of medical practice on this topic in UK have been published. Three studies have evaluated the use of pharmaceutical agents in WLWH. Two of these studies evaluated cabergoline and reported it to be an effective method of lactation inhibition in this population. The third study evaluated ethinyl estradiol and bromocriptine use and showed poor efficacy. Cabergoline is a long‐acting dopamine D2 agonist and ergot derivative that inhibits prolactin secretion and suppresses physiologic lactation when given as a single oral dose of 1 mg after delivery. Cabergoline is at least as effective as bromocriptine for lactation inhibition with success rates between 78% and 100%. Transient, mild to moderate adverse events to cabergoline are described in clinical trials. Few drug interactions exist as cabergoline is neither a substrate nor an inducer/inhibitor of hepatic cytochrome P450 isoenzymes. There are no reported clinically significant drug–drug interactions between cabergoline and any antiretroviral medications including protease inhibitors.

**Conclusions:**

Cabergoline is a safe and effective pharmacologic option for the prevention of physiological lactation and associated physical symptoms in non‐breastfeeding women. Future studies should focus on its safety, efficacy and acceptability among WLWH.

## 1 Introduction

HIV is present in breast milk in both cell‐free and cell‐associated (i.e. intracellular HIV DNA) compartments and transmission of HIV from a mother to her infant has been well documented 1. The risk of HIV transmission through breastfeeding is estimated to be at least 16% in antiretroviral naïve mothers but may be as high as 29% in the setting of primary HIV infection 1, 2. In the presence of maternal combination antiretroviral drug therapy (cART) and exclusive breastfeeding, postnatal transmission at 12 months of age has been reported to be less than 3% 3, 4. However, because maternal cART is likely to reduce only cell‐free, and not cell‐associated virus, a risk of transmission may still exist 5 and indeed, HIV transmission has been reported despite undetectable viral load in maternal plasma and breast milk 6, 7.

In high‐income countries, including Canada, the United States (US) and the United Kingdom (UK) where safe alternatives to breast milk are available, exclusive formula feeding is recommended for all infants who are born to women living with HIV (WLWH) regardless of antiretroviral drug therapy regimen and/or HIV viral load 5, 8, 9, 10, 11. Some harm reduction strategies have been recommended recently to accommodate women who choose to breastfeed despite intensive counselling 5, 10, 11. However, for women who choose not to breastfeed, the recommendations about lactation inhibition for WLWH are scarce.

A wide range of non‐pharmacologic methods are used for immediate inhibition of lactation after birth, however, a 2012 systematic review concluded that there was no evidence to indicate whether non‐pharmacologic approaches are more effective than no treatment for lactation suppression 12. Pharmacologic agents for lactation inhibition were commonly employed from 1930 to the late 1980s 13. These agents typically included oestrogen preparations and the dopamine agonist/ergot derivative, bromocriptine. However, as a result of rare but potential serious and/or fatal adverse cardiovascular, neurological and psychiatric effects bromocriptine is no longer indicated for lactation inhibition 14, 15, 16.

In the early 1990s, cabergoline, a new dopamine agonist/ergot derivative with unique pharmacokinetic properties that differentiated it from all other dopamine agonists became available 17. Early data suggested that cabergoline had similar efficacy as bromocriptine in inhibiting lactation with the advantages of easier dosing, better tolerability and fewer drug interactions 17. Currently, cabergoline is considered the first‐line therapy for patients with pituitary prolactinomas 18, and in several countries including Canada 19, the UK 20, 21 and France 22, (but not the US 23), it has an approved indication for the prevention of physiological lactation in the puerperium for clearly defined medical reasons.

The purpose of this paper is twofold: 1) To perform a scoping review regarding post‐partum pharmaceutical lactation inhibition for WLWH; 2) To summarize the available data on using cabergoline for the purpose of post‐partum lactation inhibition by reviewing its pharmacology, clinical trial efficacy and safety data, and evaluating its potential for interactions with ART.

## 2 Methods

This scoping review was performed following guidelines of the International Journal of Evidence‐Based Healthcare 24 and registered on Prospero (108902).

### 2.1 Information sources and search strategy

Potentially relevant studies were identified through a systematic and simultaneous electronic search in PubMed, Medline Ovid, EBM Reviews Ovid, Embase, Web of Science and Scopus. No year restriction was imposed in the electronic search and the last updated search was performed on 12 February 2019. Studies were identified in the aforementioned databases using the following four groups of search terms: (1) “lactation disorder;” OR (2) “lactation” AND (3) “suppressing agent;” AND (4) “HIV.” These groups were combined and researched in the title, abstract and keywords of the studies published by the journals/documents indexed in the searched databases. Finally, a manual search was also conducted in: (a) the reference lists of the articles and (b) the reference list of the latest version of the US HIV perinatal guidelines 5.

### 2.2 Inclusion criteria

The review was restricted to human participants and to publications in English or French. Original studies, case studies, book chapters, conference proceedings, were selected if they reported the use of drug for lactation inhibition for WLWH. Non‐original studies, such as guidelines and reviews, were selected if they provided guidance regarding lactation inhibition for WLWH.

### 2.3 Selection of the relevant studies

As recommended by the PRISMA Statement 25, the eligibility of the relevant studies was determined based on the examination of the titles–abstracts and full texts. First, the relevance of the titles–abstracts of the studies were independently screened by two authors (IB and KT). Then, the full texts of the studies selected based on their titles–abstracts were independently screened by the same authors to assess their eligibility. At each step, discrepancies between authors were resolved by discussion until an agreement was reached.

### 2.4 Data extraction

The same two authors independently extracted the information from the selected articles. The following information was extracted: (a) location (country) and year of publication; (b) design (cohort, cross‐sectional, case–control); (c) inclusion criteria; (d) sample size; (e) type/dose of lactation inhibition agent; (f) antiretroviral use; (g) efficacy and tolerance. The information and the data extracted were reviewed and discrepancies were resolved by discussion.

## 3 Results

**Figure 1:**
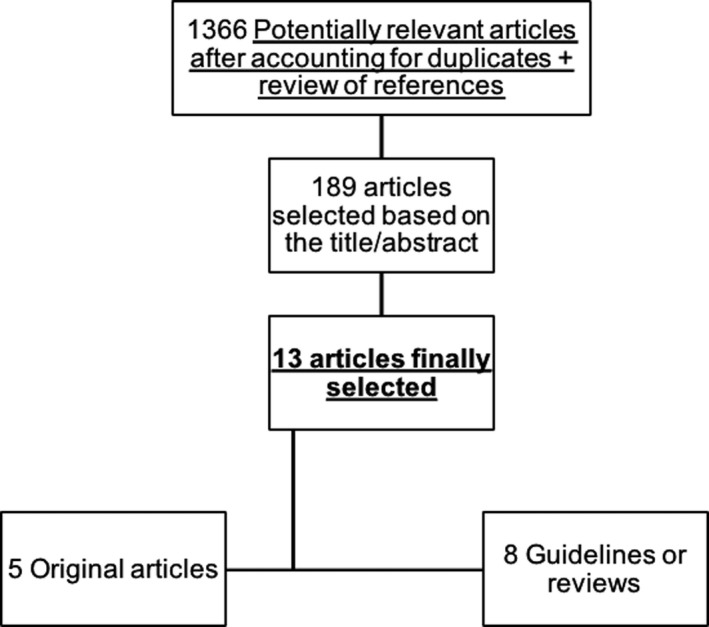
Flow chart of articles’ selection

The selection process is illustrated in Figure 1. Thirteen articles were included.

### 3.1 Guidelines and reviews

We have identified eight guidelines or reviews which provide some guidance regarding how to support breastfeeding avoidance in WLWH. While as early as 1998, De Ruiter suggested that WLWH “may benefit from the administration of bromocriptine or cabergoline” 26, there is no discussion of lactation inhibition in the US 5, the Australian 27 or the World Health Organization guidelines 28, 29. The 2014 and the 2017 Swedish guidelines suggest that “The woman should be provided support to interrupt the milk production” but no specific measure is suggested 30, 31. The 2008 British guidelines suggested for the first time the use of cabergoline 32. This was followed by recommendations from the European Agency's Pharmacovigilance Risk Assessment Committee for the use of bromocriptine “when there are compelling medical reasons for stopping lactation, such as […] in mothers with HIV infection” 15. The 2018 British guidelines recommend that women not breastfeeding their infant by choice, or because of viral load > 50 HIV RNA copies/mL, should be offered cabergoline to suppress lactation 10. While the French College of Gynaecologists and Obstetricians 33 indicated in 2016 that in the context of HIV infection, benefits and risks of pharmacological treatment to inhibit lactation must be discussed, the notion of pharmacological inhibition was not incorporated into the French HIV perinatal guidelines 34. In the 2014 Canadian guidelines 9, non‐specific measures to manage the symptoms of breast engorgement are suggested, including acetaminophen and ibuprofen. These guidelines contraindicate the co‐administration of bromocriptine or cabergoline with protease inhibitors.

In the 2009 and 2012 Cochrane reviews about lactation inhibition, there is a specific discussion on lactation inhibition to prevent HIV vertical transmission 12, 35; in these reviews it is mentioned that “The symptoms associated with physiologic cessation of lactation may further compromise the physical and emotional status of the HIV‐positive mothers and an effective method of suppressing lactation is desirable to avoid additional morbidity.”

### 3.2 Studies about lactation inhibition in WLWH

Only five studies are specifically focused on this issue; the summary of their findings is provided in Table 1. Two articles report prospective studies which found good tolerance to medication to inhibit lactation among WLWH 36, 37. However, none of these studies specified whether women were receiving cART. A retrospective pre–post study of WLWH on cART included 28 women who received cabergoline within 48 hours after delivery and 32 women who did not 38. The fourth study on this topic is a survey of healthcare workers in the UK about practices regarding lactation inhibition in WLWH 39. Follow‐up data of an UK national survey from 112 UK HIV services describing the management of pregnancy in WLWH were presented 40. Both studies showed that cabergoline is commonly used in UK for lactation inhibition in WLWH. Moreover, in a British study from 1998 about cost‐effectiveness 41, it was estimated that 40% of WLWH were prescribed bromocriptine to suppress the production of breast milk in UK, but no reference is provided to support this number.

**Table 1:** Summary of studies addressing lactation inhibition in women living with HIV

First author, year	Country	Methods	Findings
Piya‐Anant, 2004 37	Thailand	Randomized control trial n = 230 WLWH delivered at term	116 received combined pill containing 50 μg ethinyl estradiol and 114 received bromocriptine twice a day for five days. 28% of combined pills users and 25% of bromocriptine users had breast engorgement (NSD). No side effects reported.
Buhendwa, 2008 36	Malawi	Prospective cohort n = 98 WLWH who received cabergoline 1 mg at the delivery room	All women considered cabergoline effective and would like it to be available routinely. 4% minor side effects (dizziness, 2; epigastric pain, 2 lasting < 3 days).
Pammi, 2012 39	United Kingdom	Survey n = 85 healthcare workers	23% respondents routinely prescribed drugs for post‐partum lactation inhibition, and of these 75% used cabergoline. 43% respondents were aware of the interactions between antiretroviral therapy and dopaminergic lactation inhibition agents, with 22% indicating that the interactions were significant enough to avoid dopaminergic agents.
Gilleece, 2014 40	United Kingdom	Survey n = 112 HIV services	56% offer cabergoline routinely, 16% offer cabergoline in some circumstances, 19% do not use cabergoline, 8% not sure/did not answer
Humphrey 2018 38	United States	Retrospective cohort N = 28 WLWH who received cabergoline 1 mg within 48 hours post‐partum N = 32 WLWH who did not N = 164 HIV‐negative women (43 exposed to cabergoline)	94% reported effective lactation suppression post‐partum, 6% breast engorgement/leaking (46% data missing) No reported adverse effects. Cabergoline‐exposed women had lower mean systolic BPs at four‐hour time intervals within 24 hours after cabergoline without noted symptoms or increased heart rate.

NSD, non‐significant difference; WLWH, women living with HIV.

One study from Malawi reported that among 98 WLWH, all would like cabergoline to be available routinely, suggesting that they were all satisfied by the treatment 36. However, no study has directly assessed the experience/satisfaction of WLWH regarding pharmaceutical lactation inhibition nor the associated psychosocial aspects.

No pharmacokinetic data in WLWH were identified.

## 4 Discussion

As there were no published data on cabergoline pharmacology specific to WLWH, an additional narrative review was conducted to identify potentially relevant studies through an electronic search in Pubmed, Medline Ovid and Lexi‐drug Interact using the search terms “cabergoline,” “lactation inhibition” and “drug interaction.” A manual search of the reference list of identified articles was also conducted. The literature search was restricted to English language studies conducted in humans and updated until 20 February 2019. A review of the Canadian and US cabergoline product monographs was also conducted.

### 4.1 Cabergoline pharmacology

Prolactin is a hormone that is synthesized and secreted from lactotroph cells on the anterior pituitary gland, and it is responsible for the synthesis and maintenance of milk secretion. Hypothalamic control of prolactin production and release is mediated by the tonic inhibition of dopamine. Dopamine agonists act on dopamine D2‐type receptors on pituitary lactotroph membranes, leading to a decrease in the synthesis and release of prolactin 18, 42 and thereby inhibition of lactation.

Based on animal data, cabergoline is an ergoline dopamine D2 agonist with low affinity for dopamine D1‐, α‐1 and 2‐adrenergic, and 5‐HT1 and 5‐HT2 serotonergic receptors (it does have 5‐HT2B agonist activity) 19, 43. In addition, it has no effect on the basal secretion of other anterior pituitary hormones including GH, FSH, LH, corticotropin, TSH or cortisol 19. In comparison to bromocriptine, cabergoline has greater affinity for D2 receptors, and lower affinity for D1 non‐dopaminergic brain receptors 19, 43. In healthy volunteers, prolactin inhibition occurred at cabergoline doses ≥ 0.2 mg, while doses ≥ 0.5 mg caused maximal suppression in most subjects 19. Higher doses produced prolactin suppression in a greater proportion of subjects with an earlier onset and longer duration of action 19, 43.

Cabergoline pharmacokinetics has been established based on data in non‐pregnant healthy volunteers and parkinsonian patients. Cabergoline is rapidly and well absorbed with peak concentrations achieved at approximately two to three hours and is unaffected by food 19, 43. It is moderately bound to plasma proteins (41% to 42%) and has a long elimination half‐life of approximately 63 to 69 hours 19. This long elimination half‐life is the most distinctive characteristic when compared to other dopamine agonists. Cabergoline is widely distributed throughout the body and crosses the blood–brain barrier 19. It undergoes extensive hepatic metabolism primarily via hydrolysis into inactive metabolites with minimal cytochrome (CYP) P 450 enzyme involvement 19. Cabergoline pharmacokinetic parameters are unaltered in renal or mild to moderate hepatic insufficiency 19, 43. However, increased peak concentrations (C_max_) and area under the concentration curve (AUC) parameters have been observed in severe hepatic insufficiency (Child–Pugh score > 10) 19, 43. There is no evidence of any clinically significant age or gender differences in cabergoline pharmacokinetics 43.

### 4.2 Efficacy of cabergoline for lactation inhibition

Based on pharmacokinetic data, it was expected that a single cabergoline dose would be sufficient to inhibit lactation 17. Initial studies were conducted as placebo‐controlled dose‐ranging trials intended to establish an optimal dose, while larger randomized controlled trials attempted to evaluate comparative efficacy and safety. The majority of trials involved healthy women who chose not to breastfeed post‐partum for personal reasons. Table 2 contains information on all published placebo‐controlled and comparative English language trials that have evaluated cabergoline for the purpose of lactation inhibition.

**Table 2:** Summary of comparative studies about efficacy and tolerance of cabergoline for lactation inhibition

Study	Design	Population	Intervention (n)	Comparator (n)	Timing of first dose post‐ delivery (hours)	Efficacy in lactation inhibition^a^ n (%)	Efficacy in prolactin decrease	Adverse events n (%)
Melis, 1987^b^ ^44^	R, SB, PC, dose‐ranging	N = 17 healthy post‐partum women	CBG 0.4, 0.6 mg single oral dose (7/group)	PB single oral dose (5)	≤48	CBG 0.6 mg: 5 (100) CBG 0.4 mg: 3 (43) PB: 0 (0)	Serum PRL levels did not decrease in PB group (*p *>* *0.05) versus significant decrease in both CBG groups from baseline at 12 hours; reduction faster in CBG 0.6 mg group	Not reported
Melis, 1988 45	R, DB, PC, dose‐ranging	N = 32 healthy post‐partum women	CBG 0.4, 0.6, 0.8 mg single oral dose (8/group)	PB single oral dose (8)	≤24	CBG 0.8 mg: 8 (100) CBG 0.6 mg: 8 (100) CBG 0.4 mg: 4 (50) PB: 1 (13)	Serum PRL levels did not decrease in PB group versus significant decrease in all CBG groups from baseline; decreases NSD between CBG doses	0 (0)
Caballero‐Gordo, 1991 46	R, DB, PC, dose‐ranging	N = 140 healthy post‐partum women	CBG 0.5, 0.75, 1.0 mg single oral dose (40/group)	PB single oral dose (20)	≤24	*Absence of breast symptoms until 14 days:* CBG 1 mg: 36 (90) CBG 0.75 mg: 25 (63) CBG 0.5 mg: 18 (45) PB: 4 (20)	Serum PRL levels did not decrease in PB group versus significant decrease in all CBG groups from baseline; decreases NSD between CBG doses	Dizziness: 3 (3) Headache: 2 (2) Occurred with CBG 0.75 mg and 1 mg doses between one and three days; SBP/DBP changes NSD between groups
Giorda, 1991 47	R, SB	N = 36 healthy post‐partum women, caesarean section delivery	CBG 1 mg single oral dose (18)	Bromo 2.5 mg BID for 14 days (18)	≤50	*Absence of breast symptoms until 14 days:* c CBG: 15 (83) Bromo: 14 (78)	Rapid fall in serum PRL levels at three, five, seven days in both groups; increase in serum PRL at 14 days in both groups (absolute increase lower in cabergoline versus Bromo group)	CBG Dizziness: 3 (17) Headache: 1 (6) Vomit: 1 (6) Bromo Nausea: 3 (17) Dizziness: 2 (11) Headache: 2 (11) Amaurosis: 1 (6)
Rolland, 1991 49	R, DB, parallel group, MC, intention to treat	N = 272 healthy post‐partum women	CBG 1 mg single oral dose (136)	Bromo 2.5 mg BID for 14 days (136)	≤27	*Absence of breast symptoms until 15 days:* CBG: 106 (78) Bromo: 94 (69) (−8.82, 95% CI −19.25% to 1.61%) *Partial success:* d CBG, 21 (15) Bromo, 33 (24) *Failure:* d CBG, 9 (7) Bromo, 9 (7) *Rebound lactation:* e CBG, 5 (5) Bromo, 23 (24); *p *<* *0.0001	Serum PRL decreased significantly in both groups from baseline; reduction more prompt in CBG group; PRL rebound after 15 days occurred in Bromo group	CBG 18% overall rate (25 events in 22 women) Dizziness: 8 (6) Headache: 7 (5) Nausea, abdominal pain: 2 (2) Drowsiness, vertigo: 1 (<1) Orthostatic hypotensive changes:^f^ 10 (7); Symptomatic: 4 (3) Bromo 26% overall rate (44 events in 36 women) Dizziness: 17 (13) Nausea: 10 (7) Headache: 6 (4) Vomiting: 3 (2) Orthostatic hypotensive changes:^f^ 19 (14); Symptomatic: 10 (7)
Nisha, 2009^g^ ^48^	R	N = 102 healthy post‐partum women	Cabergoline 1 mg single oral dose, (54)	Combination Oestrogen‐androgen^h^ single IM injection, (48)	Not reported	CBG, 54 (100) Oestrogen‐Androgen, 47 (98); *p *=* *0.286 *Mean days for inhibition* CBG 0.73 days Oestrogen‐Androgen 1.81 days; *p *=* *0.001	Not reported	Not reported

BID, twice daily; Bromo, bromocriptine; CI, confidence interval; DBP, diastolic blood pressure; IM, intramuscular; MC, multi‐centred; NSD, not significantly different; PB, placebo; PC, placebo‐controlled; PRL, prolactin; R, randomized; SB, single‐blinded; SBP, systolic blood pressure.

^a^Efficacy variables include spontaneous milk secretion, breast engorgement, breast pain; ^b^data reported in table is for n = 17 post‐partum women enrolled; does not include data for n = 12 normal cycling controls or n = 24 hyperprolactinemic women; ^c^absence of breast pain, breast engorgement and milk secretion; ^d^partial success is presence of any moderate milk secretion, or mild to moderate breast pain, or both at 14 days, or breast engorgement at any time; failure is presence of severe breast engorgement or pain, or abundant milk secretion at end of 14‐day study period; ^e^rebound lactation is recurrence of breast symptoms measured at 16 to 21 days; ^f^drop of systolic and diastolic blood pressure of *≥* 20 mmHg; ^g^data reported in table is for n = 102 women enrolled for purpose of lactation inhibition due to stillbirth (i.e. no milk output); does not include data for n = 94 women enrolled for purpose of lactation suppression (i.e. had established lactation); ^h^estradiol benzoate (1 mg), estradiol phenyl propionate (4 mg), testosterone propionate (20 mg), testosterone phenyl propionate (40 mg), isocaproate (40 mg).

Dose‐ranging studies 44, 45, 46 have demonstrated an increased efficacy with cabergoline single oral doses from 0.4 to 1 mg, with significant decrease of serum prolactin levels compared to placebo. Findings of these early placebo‐controlled studies were then validated by three comparator trials using the highest cabergoline dose (1.0 mg) studied 39, 47, 48. The largest comparative trial was published by Rolland et al. in a prospective, randomized, double‐blind parallel group, multicentre trial with intention to treat analysis, which randomized 272 healthy post‐partum women to receive a single oral dose of cabergoline 1 mg (given within 27 hours of delivery) or bromocriptine 2.5 mg orally twice daily for 14 days 49. Women with a history of agalactia or hypogalactia, drug allergy, intrauterine foetal death, pre‐eclampsia, liver or kidney impairment and those with concomitant acute diseases were excluded from this study. Cabergoline was found to be non‐inferior to bromocriptine for lactation inhibition with a reported −8.82% point difference (90% CI −19.25% to 1.61%) for complete success (defined as the absence of breast symptomatology from day 1 to day 15), excluding the pre‐specified inferior effectiveness of cabergoline of ≥ 10%). In addition, rebound of breast symptoms and serum prolactin were reported in significantly fewer cabergoline treated women.

A recent drug use evaluation study on cabergoline use for post‐partum lactation inhibition was conducted at a women's hospital in Qatar 50. In this study 51% (n = 43) of women received cabergoline because of stillbirth, 27% (n = 23) due to pregnancy termination and 13% (n = 11) because of neonatal death. Only 9% (n = 8) of the women delivered a live infant. The majority (six of eight) of these women received cabergoline because they were receiving medications for existing medical comorbidities including renal transplant, depression, epilepsy or cardiomyopathy. Eighty‐four per cent (n = 71) of women were prescribed cabergoline within the first 27 hours of delivery. However, 50% (four of eight) of women with a live infant received cabergoline after 27 hours of delivery. The most common dose was a single oral 1 mg dose (72%, n = 61) followed by 0.25 mg twice daily for four doses (25%, n = 21).

### 4.3 Safety of cabergoline for lactation inhibition

#### 4.3.1 Adverse events

Cabergoline has been well tolerated in clinical trials when used as a single dose for inhibition of lactation with most reported side effects being transient and mild to moderate in severity (Table 2, 36, 44, 45, 46, 47, 48, 49).As per the product monograph, side effects most commonly reported include asymptomatic decreases in blood pressure, dizziness and vertigo, headache, nausea and abdominal pain [19, 23.] The maximal hypotensive effect of a single dose usually occurs during the first six hours after drug intake and is dose dependent both in terms of maximal decrease and frequency 19.

In the largest randomized controlled study comparing single dose cabergoline to bromocriptine, there were numerically fewer adverse events reported in the cabergoline versus the bromocriptine group (Table 2), however these differences were not statistically significant 49. The majority of cabergoline reported adverse events in this trial were reported on the first one to three days post‐partum and were considered mild to moderate in severity. Unexpected adverse events occurred in two women given cabergoline including epistaxis, and transient hemianopia, and one woman randomized to cabergoline stopped taking placebo on day eight due to moderate epigastric pain.

The Canadian and US product monographs list uncontrolled hypertension, a history of pulmonary, pericardial and retroperitoneal fibrotic disorders, history of cardiac valvulopathy and known hypersensitivity to cabergoline or any ergot derivative as contraindications to prescription 19, 23. In addition, the product manufacturer has issued additional warnings and precautions with using cabergoline in patients with pregnancy‐induced hypertension (e.g. pre‐eclampsia, eclampsia, post‐partum hypertension), using initial doses greater than 1 mg administering cabergoline with other medications known to lower blood pressure, or to patients with cardiovascular disease or Raynauld's syndrome as symptomatic hypotension may occur. As noted, the US monograph continues to precaution against the use of cabergoline in the setting of post‐partum lactation inhibition as a result of bromocriptine having been associated with cases of hypertension, stroke and seizures 23.

When bromocriptine was used for the inhibition of lactation there were concerns regarding the occurrence of post‐partum psychiatric reaction or relapse 51. Because the onset or relapse of psychosis is thought to be associated with dysregulation of brain dopaminergic activity and this risk appears to be greater during the early post‐partum period, it was questioned whether lactation inhibition with D2 dopamine agonists, including cabergoline, would increase this risk. A 2016 systematic review of literature published between 1950 and December 2015 identified only one single case report of cabergoline (vs. nine with bromocriptine) triggering a manic episode in a patient with a pre‐existing psychiatric illness, and none in women without pre‐existing histories of schizophrenia, bipolar disorder or pospartum psychosis 51. Based on their findings, authors concluded that while D2 agonists may increase the risk of post‐partum psychosis, bromocriptine appeared to pose a much greater risk than cabergoline.

#### 4.3.2 Drug Interactions

Cabergoline's metabolism is independent of the hepatic CYP P450 enzyme system, and it does not induce or inhibit CYP P450 isoenzymes 19. Therefore, cabergoline has not been demonstrated to interact with other medications that effect, or are affected by the hepatic CYP P450 enzymes. Cabergoline is, however, a likely P‐glycoprotein (P‐gp) transporter substrate at the blood–brain‐barrier, as demonstrated in a study showing a higher probability of central nervous system (CNS) side effects in patients with certain ABCB1 (i.e. the P‐gp gene) polymorphisms 52. It is unknown if cabergoline is an intestinal P‐glycoprotein substrate.

According to the cabergoline product monograph 19, 23, while there is no documented interaction, cabergoline should not be administered with other ergot alkaloids due to the theoretical risk of additive toxicity with long‐term treatment. In addition, concomitant administration of dopamine antagonists (e.g. metoclopramide, phenothiazines), should be avoided as co‐administration potentially reduces the prolactin‐lowering effect of cabergoline.

Two papers, describing a potential drug interaction between clarithromycin/cabergoline and itraconazole/cabergoline have been published. The first paper 53, describes improvement in parkinsonian symptoms in two patients with Parkinson's disease (PD) stabilized on long‐term cabergoline, after addition of itraconazole therapy. In one case, a 300% increase in cabergoline concentration without associated toxicity was reported, and in the second, cabergoline toxicity in the form of hyperkinesia requiring cabergoline dose reduction subsequently occurred. In the second paper, a pharmacokinetic study evaluating co‐administration of clarithromycin and cabergoline in ten healthy adults and seven patients with PD demonstrated that clarithromycin resulted in mean cabergoline concentration increases to about 2.6‐fold and 1.7‐fold in healthy volunteers and PD patients respectively 54. There was no dose‐related adverse event reported in this paper, however, three of seven PD patients experienced improvement in parkinsonian symptoms. While investigators of these reports attribute the mechanism of interaction as itraconazole/clarithromycin‐mediated inhibition of the CYP3A4 metabolism of cabergoline, it has subsequently been hypothesized to have occurred as a result of inhibition of the P‐gp transporter (a transporter which both clarithromycin and itraconazole are known to inhibit), which is responsible for the efflux of cabergoline from the CNS 53, 54, 55.

Because protease inhibitors are among the first‐line antiretroviral medications recommended for pregnant WLWH 5, 9 and most agents are strong inhibitors of the hepatic CYP 450 isoenzymes as well as the P‐gp transporter found in the intestine and blood–brain barrier 56, it is important to examine for potential drug interactions between these agents and cabergoline. Indeed, there is evidence of significant interaction between protease inhibitors and the traditional ergot derivatives (e.g. ergonovine, dihydroergotamine, ergotamine) with reports of serious adverse reactions when these ergots have been taken concomitantly with protease inhibitor‐based cART 57, 58, 59, 60, 61, 62. There are, however, no drug interaction reports between cabergoline and any antiretroviral medications, including the protease inhibitors. Similar to the interactions described between cabergoline and itraconazole and clarithromycin, any potential interaction between protease inhibitors and cabergoline would only be expected to occur through protease inhibitor‐mediated inhibition of the P‐gp substrate 56 and not the hepatic CYP P450 enzyme system. However, there is currently no evidence that a clinically important drug–drug interaction exists at this time and the concomitant use of protease inhibitors and cabergoline is not a contraindication by the product manufacturer 19, 23.

### 4.4 Limitations

There are a few limitations that have been identified in our scoping review. Only papers and abstracts that were published in English or in French were included. This especially limited our ability to extensively review existing guidelines focusing on WLWH perinatal care. We were, however, able to review several European and North American guidelines. Secondly, as there was no data specifically describing cabergoline pharmacokinetics in WLWH and/or women receiving cART, our conclusions have been extrapolated from findings in healthy volunteers and/or patients with Parkinson's Disease.

## 5 Conclusions

In most high‐income countries, where safe alternatives to breast milk are available, breastfeeding is not recommended for WLWH. Unfortunately, there are very few data and recommendations regarding lactation inhibition in this population despite the important need to provide optimal management for prevention of physical lactation symptoms in non‐breastfeeding WLWH. The dopaminergic ergot derivative, cabergoline offers an important pharmacologic option to achieve this. Compared to bromocriptine, it has similar efficacy at inhibiting lactation with associated advantages of easier dosing, fewer overall and serious side effects and fewer drug interactions. Based on our literature review, no interaction with cART is anticipated. However, while survey results indicate that HIV providers in the UK are routinely using cabergoline, there is a lack of data about cabergoline safety and efficacy in WLWH on ART. Moreover, the acceptability of drug inhibition and the psychosocial impact of breastfeeding avoidance in this context need to be considered. An ongoing prospective study in Canada is evaluating its safety, efficacy and acceptability in WLWH. Results from this, and future trials will help to address existing gaps in the literature and potentially support its incorporation into perinatal HIV guidelines.

## Competing interests

The authors declare that they have no financial or other conflicts of interest in relation to this research and its publication.

## Authors’ contributions

All persons who meet authorship criteria are listed as authors, and all authors certify that they have participated sufficiently in the work to take responsibility for the content, including participation in the concept, design, analysis, writing or revision of the manuscript. Conception and design of study: KT, IB, DM, PD, FTR. Acquisition of data: KT, IB. Analysis and interpretation of data: KT, IB, DM, CE. Drafting manuscript: KT, IB. Critical revision of manuscript: KT, IB, DM, CE, PD, FTR. All authors have read and approved the final manuscript.

## Supporting information


**Data S1.** Review strategy.
