# Supplementary material for: Cabergoline: a review of its use in the inhibition of lactation for women living with HIV
Source: J Int AIDS Soc. 2019 Jun 11;22(6):e25322. doi: 10.1002/jia2.25322 (PMC6558502; doi:10.1002/jia2.25322)
Supplement: Supplementary file 1 — Data S1. Review strategy. [file JIA2-22-e25322-s001.docx]

**Scoping review:**

**Lactation inhibition AND HIV**

[PubMed 1](#_Toc489543407)

[Medline Ovid 4](#_Toc489543408)

[EBM Reviews Ovid 5](#_Toc489543409)

[Embase 6](#_Toc489543410)

[Web of Science 7](#_Toc489543411)

[Scopus 8](#_Toc489543412)

## PubMed

| 1 | Lactation disorders | Lactation Disorders[MH] OR ((lactation*[TIAB] OR hypogalactia*[TIAB] OR colostrum*[TIAB] OR milk*[TIAB] OR breastmilk*[TIAB] OR breastfeed*[TIAB] OR breastfed*[TIAB] OR breast feed*[TIAB] OR breast fed*[TIAB] OR lactation*[OT] OR hypogalactia*[OT] OR colostrum*[OT] OR milk*[OT] OR breastmilk*[OT] OR breastfeed*[OT] OR breastfed*[OT] OR breast feed*[OT] OR breast fed*[OT]) AND (block*[TIAB] OR inhibit*[TIAB] OR suppress*[TIAB] OR interrupt*[TIAB] OR block*[OT] OR inhibit*[OT] OR suppress*[OT] OR interrupt*[OT])) |
| --- | --- | --- |
| 2 | Lactation | Lactation[MH] OR Colostrum[MH] OR Milk, Human[MH] OR Breast Feeding[MH] OR lactation*[TIAB] OR hypogalactia*[TIAB] OR colostrum*[TIAB] OR milk*[TIAB] OR breastmilk*[TIAB] OR breastfeed*[TIAB] OR breastfed*[TIAB] OR breast feed*[TIAB] OR breast fed*[TIAB] OR lactation*[OT] OR hypogalactia*[OT] OR colostrum*[OT] OR milk*[OT] OR breastmilk*[OT] OR breastfeed*[OT] OR breastfed*[OT] OR breast feed*[OT] OR breast fed*[OT] |
| 3 | Inhibition drugs | Bromocriptine[MH] OR 2 bromo alpha ergokriptin*[TIAB] OR 2 bromo alpha ergokryptin*[TIAB] OR 2 bromo alpha ergocriptin*[TIAB] OR 2 bromo alpha ergocryptin*[TIAB] OR antipark*[TIAB] OR bromed*[TIAB] OR bromergon*[TIAB] OR bromocriptin*[TIAB] OR bromocryptin*[TIAB] OR bromoergocriptin*[TIAB] OR bromoergocryptin*[TIAB] OR bromokin*[TIAB] OR bromopar*[TIAB] OR cb154*[TIAB] OR "cb 154"[TIAB] OR ergobromocriptin*[TIAB] OR ergoset*[TIAB] OR parlodel*[TIAB] OR serocryptin*[TIAB] OR cabergoline [Supplementary Concept] OR actualene*[TIAB] OR cabarsuss*[TIAB] OR cabaser*[TIAB] OR cabergolin*[TIAB] OR cabest*[TIAB] OR dostinex*[TIAB] OR fce 21336*[TIAB] OR fce21336*[TIAB] OR galastop*[TIAB] OR sogilen*[TIAB] OR sostilar*[TIAB] OR velactis*[TIAB] OR Diethylstilbestrol[MH:NOEXP] OR acnestrol*[TIAB] OR agostilben*[TIAB] OR "alpha,alpha' diethyl 4,4' dihydroxybibenzyl"[TIAB] OR "alpha,alpha' diethyl 4,4' stilbenediol"[TIAB] OR apstil*[TIAB] OR boestrol*[TIAB] OR clinestrol*[TIAB] OR clinoestrol*[TIAB] OR comestrol*[TIAB] OR cycloestrol*[TIAB] OR cyren a*[TIAB] OR "d.e.s."[TIAB] OR "di estryl"[TIAB] OR diaethylstilboestrol*[TIAB] OR diethylbestrol*[TIAB] OR diethylstilbestrol*[TIAB] OR diethylstilboestrol*[TIAB] OR dihydrostilbestrol*[TIAB] OR dihydrostilboestrol*[TIAB] OR distilbene*[TIAB] OR domestrol*[TIAB] OR esoestrol*[TIAB] OR estimon*[TIAB] OR estrastilben*[TIAB] OR estrene*[TIAB] OR estrobene*[TIAB] OR estromenin*[TIAB] OR estromonum*[TIAB] OR estrostilben*[TIAB] OR estrosyn*[TIAB] OR ethylstilbestrol*[TIAB] OR ethylstilboestrol*[TIAB] OR folone*[TIAB] OR fonatol*[TIAB] OR grafestrol*[TIAB] OR hexanestrol*[TIAB] OR hexanoestrol*[TIAB] OR hexestrofen*[TIAB] OR hexital*[TIAB] OR hexron*[TIAB] OR "hexypheen"[TIAB] OR "hi bestrol"[TIAB] OR hormoestrol*[TIAB] OR makarol*[TIAB] OR menostilbene*[TIAB] OR "meso hexestrol"[TIAB] OR mesohexestrol*[TIAB] OR micrest*[TIAB] OR microest*[TIAB] OR mikarol*[TIAB] OR milestrol*[TIAB] OR neo estranol*[TIAB] OR "nsc 3070"[TIAB] OR nsc3070*[TIAB] OR "nsc 35752"[TIAB] OR nsc35752*[TIAB] OR "nsc 9894"[TIAB] OR nsc9894*[TIAB] OR oekolp*[TIAB] OR oestramon*[TIAB] OR oestrogeni*[TIAB] OR oestromenin*[TIAB] OR oestromensyl*[TIAB] OR oestrostilben*[TIAB] OR oestrosyntal*[TIAB] OR orestrol*[TIAB] OR oroestron*[TIAB] OR ovendosyn*[TIAB] OR pabestrol*[TIAB] OR pabestrosalve*[TIAB] OR palestrol*[TIAB] OR proestrin*[TIAB] OR serral*[TIAB] OR sinestrol*[TIAB] OR "stil rol"[TIAB] OR stilbarol*[TIAB] OR stilbenol*[TIAB] OR stilbesterol*[TIAB] OR stilbestro*[TIAB] OR stilbetin*[TIAB] OR stilboefral*[TIAB] OR stilboestro*[TIAB] OR stilbol*[TIAB] OR stilkap*[TIAB] OR synestrin*[TIAB] OR syntex*[TIAB] OR synthoestrin*[TIAB] OR synthovo*[TIAB] OR syntrogene*[TIAB] OR tampovagan*[TIAB] OR Lisuride[MH] OR arolac*[TIAB] OR cuvalit*[TIAB] OR dopergin*[TIAB] OR lisurid*[TIAB] OR lysurid*[TIAB] OR lysenyl*[TIAB] OR "methylergol carbamide"[TIAB] OR nenad*[TIAB] OR prolacam*[TIAB] OR revanil*[TIAB] OR Dihydroergocryptine[MH] OR almirid*[TIAB] OR cripar*[TIAB] OR "dhk 135"[TIAB] OR dhk135*[TIAB] OR dihydro alpha ergocryptine*[TIAB] OR dihydroergocriptin*[TIAB] OR dihydroergocryptin*[TIAB] OR dihydroergokriptin*[TIAB] OR dihydroergokryptin*[TIAB] OR myrol*[TIAB] OR vasobral*[TIAB] OR Ergot Alkaloids[MH:NOEXP] OR "clavine alkaloid"[TIAB] OR "clavine alkaloids"[TIAB] OR ergot alkaloid*[TIAB] OR "ergot drug"[TIAB] OR "ergot drugs"[TIAB] OR "ergotoxine alkaloid"[TIAB] OR Ergolines[MH:NOEXP] OR ergoline*[TIAB] OR Ergotamines[MH:NOEXP] OR ergotamine*[TIAB] OR Estradiol[MH:NOEXP] OR acrofol*[TIAB] OR aerodiol*[TIAB] OR almediol*[TIAB] OR alora*[TIAB] OR altrad*[TIAB] OR aquadiol*[TIAB] OR aquiol*[TIAB] OR bardiol*[TIAB] OR beta estradiol*[TIAB] OR "bio e gel"[TIAB] OR bisteron*[TIAB] OR cerella*[TIAB] OR climaderm*[TIAB] OR climara*[TIAB] OR climera*[TIAB] OR "clinagen la 40"[TIAB] OR compudose*[TIAB] OR "corpa gen"[TIAB] OR corpagen*[TIAB] OR delidose*[TIAB] OR "dep gynogen"[TIAB] OR dermestril*[TIAB] OR dihydro alpha follikelhormon*[TIAB] OR "dihydro oestron"[TIAB] OR dihydrofollicul*[TIAB] OR dihydromenformon*[TIAB] OR dihydrooestron*[TIAB] OR dihydrotheelin*[TIAB] OR dihydroxyestrin*[TIAB] OR dimenformon*[TIAB] OR diogyn*[TIAB] OR divigel*[TIAB] OR "e 2"[TIAB] OR elestrin*[TIAB] OR endofollicolin*[TIAB] OR "ent estradiol"[TIAB] OR esclim*[TIAB] OR estovite*[TIAB] OR estrace*[TIAB] OR estradiol*[TIAB] OR estradot*[TIAB] OR estraguard*[TIAB] OR "estragyn la 5"[TIAB] OR estraldine*[TIAB] OR estran*[TIAB] OR estrasorb*[TIAB] OR estratab*[TIAB] OR estredox*[TIAB] OR estreva*[TIAB] OR estrifam*[TIAB] OR estring*[TIAB] OR estro-cyp*[TIAB] OR estrofem*[TIAB] OR estroform*[TIAB] OR estrogel*[TIAB] OR estrogil*[TIAB] OR estrogyne*[TIAB] OR estrolan[TIAB] OR estrovite*[TIAB] OR evafilm*[TIAB] OR evamist*[TIAB] OR evepia*[TIAB] OR evorel*[TIAB] OR "fem 7"[TIAB] OR fematrix*[TIAB] OR femestral*[TIAB] OR femestrol*[TIAB] OR femiderm*[TIAB] OR femogen[TIAB] OR fempatch*[TIAB] OR femsept*[TIAB] OR femseven*[TIAB] OR femtran*[TIAB] OR folanyl*[TIAB] OR "follicormon erba"[TIAB] OR follicyclin*[TIAB] OR ginedisc*[TIAB] OR gynergon*[TIAB] OR gynodiol*[TIAB] OR gynoestryl*[TIAB] OR gynokadin*[TIAB] OR gynpolar*[TIAB] OR innofem*[TIAB] OR lamdiol*[TIAB] OR lindisc*[TIAB] OR "linoladiol n"[TIAB] OR lipoide*[TIAB] OR "lut ovocycline"[TIAB] OR lyrelle*[TIAB] OR macrodiol*[TIAB] OR "menaval-20"[TIAB] OR meno-mpa*[TIAB] OR “menodin tts”[TIAB] OR menorest*[TIAB] OR menoring*[TIAB] OR menostar*[TIAB] OR mestrace*[TIAB] OR minivelle*[TIAB] OR "nsc 20293"[TIAB] OR nsc20293*[TIAB] OR oesclim*[TIAB] OR oestergon*[TIAB] OR oestradiol*[TIAB] OR oestring*[TIAB] OR oestrodose*[TIAB] OR oestroform*[TIAB] OR oestrogel*[TIAB] OR oestroglandol*[TIAB] OR ormogamma*[TIAB] OR ovahormon*[TIAB] OR ovasterol*[TIAB] OR ovociclin*[TIAB] OR ovocyclin*[TIAB] OR ovocylin*[TIAB] OR perlatanol*[TIAB] OR primofol*[TIAB] OR primogyn*[TIAB] OR profolid*[TIAB] OR profoliol*[TIAB] OR proginon*[TIAB] OR progynon*[TIAB] OR riselle*[TIAB] OR sandrena*[TIAB] OR "sisare gel"[TIAB] OR syndiol*[TIAB] OR systen*[TIAB] OR thais*[TIAB] OR tradelia*[TIAB] OR vagifem*[TIAB] OR valergen*[TIAB] OR vivelle*[TIAB] OR vivelledot*[TIAB] OR zumenon*[TIAB] OR Estrogens[MH:NOEXP] OR estrogen*[TIAB] OR oestrogen*[TIAB] OR "kober chromogen"[TIAB] OR Ethinyl Estradiol[MH:NOEXP] OR aethinylstradiol*[TIAB] OR "diogyn e"[TIAB] OR diolyn*[TIAB] OR dyloform*[TIAB] OR "e 4876"[TIAB] OR e4876*[TIAB] OR esteed*[TIAB] OR estigyn*[TIAB] OR estinyl*[TIAB] OR "eston e"[TIAB] OR estoral*[TIAB] OR estrasal*[TIAB] OR estronex[TIAB] OR “estroral pabyrn”[TIAB] OR ethidol*[TIAB] OR ethin oestryl*[TIAB] OR ethinoral*[TIAB] OR ethinyl estradiol*[TIAB] OR ethinyl oestradiol*[TIAB] OR ethinylestradiol*[TIAB] OR ethinyloestradiol*[TIAB] OR "ethynil estradiol"[TIAB] OR ethynilestradiol*[TIAB] OR ethynyl estradiol*[TIAB] OR ethynylestra*[TIAB] OR eticiclin*[TIAB] OR eticyclin*[TIAB] OR eticyclol*[TIAB] OR eticylol*[TIAB] OR etifollin*[TIAB] OR etinestrol*[TIAB] OR etinestryl*[TIAB] OR etinilestradiol*[TIAB] OR etinoestryl*[TIAB] OR etinylestradiol*[TIAB] OR etivex*[TIAB] OR feminone*[TIAB] OR fodinyl*[TIAB] OR follicoral*[TIAB] OR follikoral*[TIAB] OR ginestrene*[TIAB] OR ginormon*[TIAB] OR gynoral*[TIAB] OR hewestrol*[TIAB] OR hewoestrol*[TIAB] OR ibioculine*[TIAB] OR inestra*[TIAB] OR kolpolyn*[TIAB] OR linoral*[TIAB] OR lynestoral*[TIAB] OR lynoral*[TIAB] OR manodiol*[TIAB] OR menolyn*[TIAB] OR metrociclina*[TIAB] OR metroval*[TIAB] OR microfollin*[TIAB] OR mikrofollin*[TIAB] OR nadestryl*[TIAB] OR neo-estrone*[TIAB] OR neo-oestrone*[TIAB] OR neoestrone*[TIAB] OR "norma oestren"[TIAB] OR novestrol*[TIAB] OR "nsc 10973"[TIAB] OR nsc10973*[TIAB] OR nylestin*[TIAB] OR oestralyn*[TIAB] OR oestroperos*[TIAB] OR oradiol*[TIAB] OR orestralyn*[TIAB] OR ostral*[TIAB] OR perovex*[TIAB] OR "primogyn c"[TIAB] OR "primogyn m"[TIAB] OR "progynon c"[TIAB] OR "progynon m"[TIAB] OR Methylergonovine[MH] OR methylergobasin*[TIAB] OR methylergobrevin*[TIAB] OR methylergonovin*[TIAB] OR methylergometrin*[TIAB] OR methergin*[TIAB] OR myomergin*[TIAB] OR 2 bromo alpha ergokriptin*[OT] OR 2 bromo alpha ergokryptin*[OT] OR 2 bromo alpha ergocriptin*[OT] OR 2 bromo alpha ergocryptin*[OT] OR antipark*[OT] OR bromed*[OT] OR bromergon*[OT] OR bromocriptin*[OT] OR bromocryptin*[OT] OR bromoergocriptin*[OT] OR bromoergocryptin*[OT] OR bromokin*[OT] OR bromopar*[OT] OR cb154*[OT] OR "cb 154"[OT] OR ergobromocriptin*[OT] OR ergoset*[OT] OR parlodel*[OT] OR serocryptin*[OT] OR actualene*[OT] OR cabarsuss*[OT] OR cabaser*[OT] OR cabergolin*[OT] OR cabest*[OT] OR dostinex*[OT] OR fce 21336*[OT] OR fce21336*[OT] OR galastop*[OT] OR sogilen*[OT] OR sostilar*[OT] OR velactis*[OT] OR acnestrol*[OT] OR agostilben*[OT] OR "alpha,alpha' diethyl 4,4' dihydroxybibenzyl"[OT] OR "alpha,alpha' diethyl 4,4' stilbenediol"[OT] OR apstil*[OT] OR boestrol*[OT] OR clinestrol*[OT] OR clinoestrol*[OT] OR comestrol*[OT] OR cycloestrol*[OT] OR cyren a*[OT] OR "d.e.s."[OT] OR "di estryl"[OT] OR diaethylstilboestrol*[OT] OR diethylbestrol*[OT] OR diethylstilbestrol*[OT] OR diethylstilboestrol*[OT] OR dihydrostilbestrol*[OT] OR dihydrostilboestrol*[OT] OR distilbene*[OT] OR domestrol*[OT] OR esoestrol*[OT] OR estimon*[OT] OR estrastilben*[OT] OR estrene*[OT] OR estrobene*[OT] OR estromenin*[OT] OR estromonum*[OT] OR estrostilben*[OT] OR estrosyn*[OT] OR ethylstilbestrol*[OT] OR ethylstilboestrol*[OT] OR folone*[OT] OR fonatol*[OT] OR grafestrol*[OT] OR hexanestrol*[OT] OR hexanoestrol*[OT] OR hexestrofen*[OT] OR hexital*[OT] OR hexron*[OT] OR "hexypheen"[OT] OR "hi bestrol"[OT] OR hormoestrol*[OT] OR makarol*[OT] OR menostilbene*[OT] OR "meso hexestrol"[OT] OR mesohexestrol*[OT] OR micrest*[OT] OR microest*[OT] OR mikarol*[OT] OR milestrol*[OT] OR neo estranol*[OT] OR "nsc 3070"[OT] OR nsc3070*[OT] OR "nsc 35752"[OT] OR nsc35752*[OT] OR "nsc 9894"[OT] OR nsc9894*[OT] OR oekolp*[OT] OR oestramon*[OT] OR oestrogeni*[OT] OR oestromenin*[OT] OR oestromensyl*[OT] OR oestrostilben*[OT] OR oestrosyntal*[OT] OR orestrol*[OT] OR oroestron*[OT] OR ovendosyn*[OT] OR pabestrol*[OT] OR pabestrosalve*[OT] OR palestrol*[OT] OR proestrin*[OT] OR serral*[OT] OR sinestrol*[OT] OR "stil rol"[OT] OR stilbarol*[OT] OR stilbenol*[OT] OR stilbesterol*[OT] OR stilbestro*[OT] OR stilbetin*[OT] OR stilboefral*[OT] OR stilboestro*[OT] OR stilbol*[OT] OR stilkap*[OT] OR synestrin*[OT] OR syntex*[OT] OR synthoestrin*[OT] OR synthovo*[OT] OR syntrogene*[OT] OR tampovagan*[OT] OR arolac*[OT] OR cuvalit*[OT] OR dopergin*[OT] OR lisurid*[OT] OR lysurid*[OT] OR lysenyl*[OT] OR "methylergol carbamide"[OT] OR nenad*[OT] OR prolacam*[OT] OR revanil*[OT] OR almirid*[OT] OR cripar*[OT] OR "dhk 135"[OT] OR dhk135*[OT] OR dihydro alpha ergocryptine*[OT] OR dihydroergocriptin*[OT] OR dihydroergocryptin*[OT] OR dihydroergokriptin*[OT] OR dihydroergokryptin*[OT] OR myrol*[OT] OR vasobral*[OT] OR "clavine alkaloid"[OT] OR "clavine alkaloids"[OT] OR ergot alkaloid*[OT] OR "ergot drug"[OT] OR "ergot drugs"[OT] OR "ergotoxine alkaloid"[OT] OR ergoline*[OT] OR ergotamine*[OT] OR acrofol*[OT] OR aerodiol*[OT] OR almediol*[OT] OR alora*[OT] OR altrad*[OT] OR aquadiol*[OT] OR aquiol*[OT] OR bardiol*[OT] OR beta estradiol*[OT] OR "bio e gel"[OT] OR bisteron*[OT] OR cerella*[OT] OR climaderm*[OT] OR climara*[OT] OR climera*[OT] OR "clinagen la 40"[OT] OR compudose*[OT] OR "corpa gen"[OT] OR corpagen*[OT] OR delidose*[OT] OR "dep gynogen"[OT] OR dermestril*[OT] OR dihydro alpha follikelhormon*[OT] OR "dihydro oestron"[OT] OR dihydrofollicul*[OT] OR dihydromenformon*[OT] OR dihydrooestron*[OT] OR dihydrotheelin*[OT] OR dihydroxyestrin*[OT] OR dimenformon*[OT] OR diogyn*[OT] OR divigel*[OT] OR "e 2"[OT] OR elestrin*[OT] OR endofollicolin*[OT] OR "ent estradiol"[OT] OR esclim*[OT] OR estovite*[OT] OR estrace*[OT] OR estradiol*[OT] OR estradot*[OT] OR estraguard*[OT] OR "estragyn la 5"[OT] OR estraldine*[OT] OR estran*[OT] OR estrasorb*[OT] OR estratab*[OT] OR estredox*[OT] OR estreva*[OT] OR estrifam*[OT] OR estring*[OT] OR estro-cyp*[OT] OR estrofem*[OT] OR estroform*[OT] OR estrogel*[OT] OR estrogil*[OT] OR estrogyne*[OT] OR estrolan[OT] OR estrovite*[OT] OR evafilm*[OT] OR evamist*[OT] OR evepia*[OT] OR evorel*[OT] OR "fem 7"[OT] OR fematrix*[OT] OR femestral*[OT] OR femestrol*[OT] OR femiderm*[OT] OR femogen[OT] OR fempatch*[OT] OR femsept*[OT] OR femseven*[OT] OR femtran*[OT] OR folanyl*[OT] OR "follicormon erba"[OT] OR follicyclin*[OT] OR ginedisc*[OT] OR gynergon*[OT] OR gynodiol*[OT] OR gynoestryl*[OT] OR gynokadin*[OT] OR gynpolar*[OT] OR innofem*[OT] OR lamdiol*[OT] OR lindisc*[OT] OR "linoladiol n"[OT] OR lipoide*[OT] OR "lut ovocycline"[OT] OR lyrelle*[OT] OR macrodiol*[OT] OR "menaval-20"[OT] OR meno-mpa*[OT] OR “menodin tts”[OT] OR menorest*[OT] OR menoring*[OT] OR menostar*[OT] OR mestrace*[OT] OR minivelle*[OT] OR "nsc 20293"[OT] OR nsc20293*[OT] OR oesclim*[OT] OR oestergon*[OT] OR oestradiol*[OT] OR oestring*[OT] OR oestrodose*[OT] OR oestroform*[OT] OR oestrogel*[OT] OR oestroglandol*[OT] OR ormogamma*[OT] OR ovahormon*[OT] OR ovasterol*[OT] OR ovociclin*[OT] OR ovocyclin*[OT] OR ovocylin*[OT] OR perlatanol*[OT] OR primofol*[OT] OR primogyn*[OT] OR profolid*[OT] OR profoliol*[OT] OR proginon*[OT] OR progynon*[OT] OR riselle*[OT] OR sandrena*[OT] OR "sisare gel"[OT] OR syndiol*[OT] OR systen*[OT] OR thais*[OT] OR tradelia*[OT] OR vagifem*[OT] OR valergen*[OT] OR vivelle*[OT] OR vivelledot*[OT] OR zumenon*[OT] OR estrogen*[OT] OR oestrogen*[OT] OR "kober chromogen"[OT] OR aethinylstradiol*[OT] OR "diogyn e"[OT] OR diolyn*[OT] OR dyloform*[OT] OR "e 4876"[OT] OR e4876*[OT] OR esteed*[OT] OR estigyn*[OT] OR estinyl*[OT] OR "eston e"[OT] OR estoral*[OT] OR estrasal*[OT] OR estronex[OT] OR “estroral pabyrn”[OT] OR ethidol*[OT] OR ethin oestryl*[OT] OR ethinoral*[OT] OR ethinyl estradiol*[OT] OR ethinyl oestradiol*[OT] OR ethinylestradiol*[OT] OR ethinyloestradiol*[OT] OR "ethynil estradiol"[OT] OR ethynilestradiol*[OT] OR ethynyl estradiol*[OT] OR ethynylestra*[OT] OR eticiclin*[OT] OR eticyclin*[OT] OR eticyclol*[OT] OR eticylol*[OT] OR etifollin*[OT] OR etinestrol*[OT] OR etinestryl*[OT] OR etinilestradiol*[OT] OR etinoestryl*[OT] OR etinylestradiol*[OT] OR etivex*[OT] OR feminone*[OT] OR fodinyl*[OT] OR follicoral*[OT] OR follikoral*[OT] OR ginestrene*[OT] OR ginormon*[OT] OR gynoral*[OT] OR hewestrol*[OT] OR hewoestrol*[OT] OR ibioculine*[OT] OR inestra*[OT] OR kolpolyn*[OT] OR linoral*[OT] OR lynestoral*[OT] OR lynoral*[OT] OR manodiol*[OT] OR menolyn*[OT] OR metrociclina*[OT] OR metroval*[OT] OR microfollin*[OT] OR mikrofollin*[OT] OR nadestryl*[OT] OR neo-estrone*[OT] OR neo-oestrone*[OT] OR neoestrone*[OT] OR "norma oestren"[OT] OR novestrol*[OT] OR "nsc 10973"[OT] OR nsc10973*[OT] OR nylestin*[OT] OR oestralyn*[OT] OR oestroperos*[OT] OR oradiol*[OT] OR orestralyn*[OT] OR ostral*[OT] OR perovex*[OT] OR "primogyn c"[OT] OR "primogyn m"[OT] OR "progynon c"[OT] OR "progynon m"[OT] OR methylergobasin*[OT] OR methylergobrevin*[OT] OR methylergonovin*[OT] OR methylergometrin*[OT] OR methergin*[OT] OR myomergin*[OT] |
| 4 | HIV | HIV[MH] OR HIV Infections[MH] OR (AIDS[TIAB] NOT (decision aids[TIAB] OR hearing aids[tiab] OR Technological aids[tiab] OR communication aids[tiab])) OR HIV[TIAB] OR acquired immunologic deficiency syndrome*[TIAB] OR acquired immune deficiency syndrome*[TIAB] OR acquired immuno deficiency syndrome*[TIAB] OR acquired immunodeficiency syndrome*[TIAB] OR human immunologic deficiency virus*[TIAB] OR human immune deficiency virus*[TIAB] OR human immuno deficiency virus*[TIAB] OR human immunodeficiency virus*[TIAB] OR immunologic deficiency associated virus*[TIAB] OR immune deficiency associated virus*[TIAB] OR immuno deficiency associated virus*[TIAB] OR immunodeficiency associated virus*[TIAB] OR (AIDS[OT] NOT (decision aids[OT] OR hearing aids[OT] OR Technological aids[OT] OR communication aids[OT])) OR HIV[OT] OR acquired immunologic deficiency syndrome*[OT] OR acquired immune deficiency syndrome*[OT] OR acquired immuno deficiency syndrome*[OT] OR acquired immunodeficiency syndrome*[OT] OR human immunologic deficiency virus*[OT] OR human immune deficiency virus*[OT] OR human immuno deficiency virus*[OT] OR human immunodeficiency virus*[OT] OR immunologic deficiency associated virus*[OT] OR immune deficiency associated virus*[OT] OR immuno deficiency associated virus*[OT] OR immunodeficiency associated virus*[OT] |
| 5 | NOT | (Animals[MH] NOT Humans[MH]) |
| 6 | Combinaison and limitations | (((((#1 OR (#2 AND #3)) AND #4) NOT #5)) AND (english[LA] OR french[LA])) = 306 résultats |

## Medline Ovid

| 1 | Lactation disorders | Exp Lactation Disorders/ OR ((lactation* OR hypogalactia* OR colostrum* OR milk* OR breastmilk* OR breastfeed* OR breastfed* OR breast feed* OR breast fed*) AND (block* OR inhibit* OR suppress* OR interrupt*)).ti,ab,kw,kf |
| --- | --- | --- |
| 2 | Lactation | exp Lactation/ OR Colostrum/ OR milk, human/ OR exp Breast Feeding/ OR (lactation* OR hypogalactia* OR colostrum* OR milk* OR breastmilk* OR breastfeed* OR breastfed* OR breast feed* OR breast fed*).ti,ab,kw,kf |
| 3 | Inhibition drugs | Bromocriptine/ OR Diethylstilbestrol/ OR Lisuride/ OR Dihydroergocryptine/ OR Ergot Alkaloids/ OR Ergolines/ OR Ergotamines/ OR Estradiol/ OR Estrogens/ OR Ethinyl Estradiol/ OR Methylergonovine/ OR (2 bromo alpha ergokriptin* OR 2 bromo alpha ergokryptin* OR 2 bromo alpha ergocriptin* OR 2 bromo alpha ergocryptin* OR antipark* OR bromed* OR bromergon* OR bromocriptin* OR bromocryptin* OR bromoergocriptin* OR bromoergocryptin* OR bromokin* OR bromopar* OR cb154* OR "cb 154" OR ergobromocriptin* OR ergoset* OR parlodel* OR serocryptin* OR actualene* OR cabarsuss* OR cabaser* OR cabergolin* OR cabest* OR dostinex* OR fce 21336* OR fce21336* OR galastop* OR sogilen* OR sostilar* OR velactis* OR acnestrol* OR agostilben* OR "alpha,alpha' diethyl 4,4' dihydroxybibenzyl" OR "alpha,alpha' diethyl 4,4' stilbenediol" OR apstil* OR boestrol* OR clinestrol* OR clinoestrol* OR comestrol* OR cycloestrol* OR cyren a* OR "d.e.s." OR "di estryl" OR diaethylstilboestrol* OR diethylbestrol* OR diethylstilbestrol* OR diethylstilboestrol* OR dihydrostilbestrol* OR dihydrostilboestrol* OR distilbene* OR domestrol* OR esoestrol* OR estimon* OR estrastilben* OR estrene* OR estrobene* OR estromenin* OR estromonum* OR estrostilben* OR estrosyn* OR ethylstilbestrol* OR ethylstilboestrol* OR folone* OR fonatol* OR grafestrol* OR hexanestrol* OR hexanoestrol* OR hexestrofen* OR hexital* OR hexron* OR "hexypheen" OR "hi bestrol" OR hormoestrol* OR makarol* OR menostilbene* OR "meso hexestrol" OR mesohexestrol* OR micrest* OR microest* OR mikarol* OR milestrol* OR neo estranol* OR "nsc 3070" OR nsc3070* OR "nsc 35752" OR nsc35752* OR "nsc 9894" OR nsc9894* OR oekolp* OR oestramon* OR oestrogeni* OR oestromenin* OR oestromensyl* OR oestrostilben* OR oestrosyntal* OR orestrol* OR oroestron* OR ovendosyn* OR pabestrol* OR pabestrosalve* OR palestrol* OR proestrin* OR serral* OR sinestrol* OR "stil rol" OR stilbarol* OR stilbenol* OR stilbesterol* OR stilbestro* OR stilbetin* OR stilboefral* OR stilboestro* OR stilbol* OR stilkap* OR synestrin* OR syntex* OR synthoestrin* OR synthovo* OR syntrogene* OR tampovagan* OR arolac* OR cuvalit* OR dopergin* OR lisurid* OR lysurid* OR lysenyl* OR "methylergol carbamide" OR nenad* OR prolacam* OR revanil* OR almirid* OR cripar* OR "dhk 135" OR dhk135 OR dihydro alpha ergocryptine* OR dihydroergocriptin* OR dihydroergocryptin* OR dihydroergokriptin* OR dihydroergokryptin* OR myrol* OR vasobral* OR "clavine alkaloid" OR "clavine alkaloids" OR ergot alkaloid* OR "ergot drug" OR "ergot drugs" OR "ergotoxine alkaloid" OR ergoline* OR ergotamine* OR acrofol* OR aerodiol* OR almediol* OR alora* OR altrad* OR aquadiol* OR aquiol* OR bardiol* OR beta estradiol* OR "bio e gel" OR bisteron* OR cerella* OR climaderm* OR climara* OR climera* OR "clinagen la 40" OR compudose* OR "corpa gen" OR corpagen* OR delidose* OR "dep gynogen" OR dermestril* OR dihydro alpha follikelhormon* OR "dihydro oestron" OR dihydrofollicul* OR dihydromenformon* OR dihydrooestron* OR dihydrotheelin* OR dihydroxyestrin* OR dimenformon* OR diogyn* OR divigel* OR "e 2" OR elestrin* OR endofollicolin* OR "ent estradiol" OR esclim* OR estovite* OR estrace* OR estradiol* OR estradot* OR estraguard* OR "estragyn la 5" OR estraldine* OR estran* OR estrasorb* OR estratab* OR estredox* OR estreva* OR estrifam* OR estring* OR estro-cyp* OR estrofem* OR estroform* OR estrogel* OR estrogil* OR estrogyne* OR estrolan OR estrovite* OR evafilm* OR evamist* OR evepia* OR evorel* OR "fem 7" OR fematrix* OR femestral* OR femestrol* OR femiderm* OR femogen OR fempatch* OR femsept* OR femseven* OR femtran* OR folanyl* OR "follicormon erba" OR follicyclin* OR ginedisc* OR gynergon* OR gynodiol* OR gynoestryl* OR gynokadin* OR gynpolar* OR innofem* OR lamdiol* OR lindisc* OR "linoladiol n" OR lipoide* OR "lut ovocycline" OR lyrelle* OR macrodiol* OR "menaval-20" OR meno-mpa* OR "menodin tts" OR menorest* OR menoring* OR menostar* OR mestrace* OR minivelle* OR "nsc 20293" OR nsc20293 OR oesclim* OR oestergon* OR oestradiol* OR oestring* OR oestrodose* OR oestroform* OR oestrogel* OR oestroglandol* OR ormogamma* OR ovahormon* OR ovasterol* OR ovociclin* OR ovocyclin* OR ovocylin* OR perlatanol* OR primofol* OR primogyn* OR profolid* OR profoliol* OR proginon* OR progynon* OR riselle* OR sandrena* OR "sisare gel" OR syndiol* OR systen* OR thais* OR tradelia* OR vagifem* OR valergen* OR vivelle* OR vivelledot* OR zumenon* OR estrogen* OR oestrogen* OR "kober chromogen" OR aethinylstradiol* OR "diogyn e" OR diolyn* OR dyloform* OR "e 4876" OR e4876* OR esteed* OR estigyn* OR estinyl* OR "eston e" OR estoral* OR estrasal* OR estronex OR "estroral pabyrn" OR ethidol* OR ethin oestryl* OR ethinoral* OR ethinyl estradiol* OR ethinyl oestradiol* OR ethinylestradiol* OR ethinyloestradiol* OR "ethynil estradiol" OR ethynilestradiol* OR ethynyl estradiol* OR ethynylestra* OR eticiclin* OR eticyclin* OR eticyclol* OR eticylol* OR etifollin* OR etinestrol* OR etinestryl* OR etinilestradiol* OR etinoestryl* OR etinylestradiol* OR etivex* OR feminone* OR fodinyl* OR follicoral* OR follikoral* OR ginestrene* OR ginormon* OR gynoral* OR hewestrol* OR hewoestrol* OR ibioculine* OR inestra* OR kolpolyn* OR linoral* OR lynestoral* OR lynoral* OR manodiol* OR menolyn* OR metrociclina* OR metroval* OR microfollin* OR mikrofollin* OR nadestryl* OR neo-estrone* OR neo-oestrone* OR neoestrone* OR "norma oestren" OR novestrol* OR "nsc 10973" OR nsc10973* OR nylestin* OR oestralyn* OR oestroperos* OR oradiol* OR orestralyn* OR ostral* OR perovex* OR "primogyn c" OR "primogyn m" OR "progynon c" OR "progynon m" OR methylergobasin* OR methylergobrevin* OR methylergonovin* OR methylergometrin* OR methergin* OR myomergin*).ti,ab,kw,kf |
| 4 | VIH | Exp HIV/ OR exp HIV infections/ OR ((AIDS NOT (decision aids OR hearing aids OR Technological aids OR communication aids)) OR HIV OR acquired immunologic deficiency syndrome* OR acquired immune deficiency syndrome* OR acquired immuno deficiency syndrome* OR acquired immunodeficiency syndrome* OR human immunologic deficiency virus* OR human immune deficiency virus* OR human immuno deficiency virus* OR human immunodeficiency virus* OR immunologic deficiency associated virus* OR immune deficiency associated virus* OR immuno deficiency associated virus* OR immunodeficiency associated virus*).ti,ab,kw,kf |
| 5 | NOT | exp animals/ not exp humans/ |
| 6 | Combinaison | (((1 OR (2 AND 3)) AND 4) NOT 5) |
| 7 | Limitation | Limit 5 to (english or french) = 463 résultats |

## EBM Reviews Ovid

| 1 | Lactation disorders | Exp Lactation Disorders/ OR ((lactation* OR hypogalactia* OR colostrum* OR milk* OR breastmilk* OR breastfeed* OR breastfed* OR breast feed* OR breast fed*) AND (block* OR inhibit* OR suppress* OR interrupt*)).ti,ab,kw,kf |
| --- | --- | --- |
| 2 | Lactation | exp Lactation/ OR Colostrum/ OR milk, human/ OR exp Breast Feeding/ OR (lactation* OR hypogalactia* OR colostrum* OR milk* OR breastmilk* OR breastfeed* OR breastfed* OR breast feed* OR breast fed*).ti,ab,kw,kf |
| 3 | Inhibition drugs | Bromocriptine/ OR Diethylstilbestrol/ OR Lisuride/ OR Dihydroergocryptine/ OR Ergot Alkaloids/ OR Ergolines/ OR Ergotamines/ OR Estradiol/ OR Estrogens/ OR Ethinyl Estradiol/ OR Methylergonovine/ OR (2 bromo alpha ergokriptin* OR 2 bromo alpha ergokryptin* OR 2 bromo alpha ergocriptin* OR 2 bromo alpha ergocryptin* OR antipark* OR bromed* OR bromergon* OR bromocriptin* OR bromocryptin* OR bromoergocriptin* OR bromoergocryptin* OR bromokin* OR bromopar* OR cb154* OR "cb 154" OR ergobromocriptin* OR ergoset* OR parlodel* OR serocryptin* OR actualene* OR cabarsuss* OR cabaser* OR cabergolin* OR cabest* OR dostinex* OR fce 21336* OR fce21336* OR galastop* OR sogilen* OR sostilar* OR velactis* OR acnestrol* OR agostilben* OR "alpha,alpha' diethyl 4,4' dihydroxybibenzyl" OR "alpha,alpha' diethyl 4,4' stilbenediol" OR apstil* OR boestrol* OR clinestrol* OR clinoestrol* OR comestrol* OR cycloestrol* OR cyren a* OR "d.e.s." OR "di estryl" OR diaethylstilboestrol* OR diethylbestrol* OR diethylstilbestrol* OR diethylstilboestrol* OR dihydrostilbestrol* OR dihydrostilboestrol* OR distilbene* OR domestrol* OR esoestrol* OR estimon* OR estrastilben* OR estrene* OR estrobene* OR estromenin* OR estromonum* OR estrostilben* OR estrosyn* OR ethylstilbestrol* OR ethylstilboestrol* OR folone* OR fonatol* OR grafestrol* OR hexanestrol* OR hexanoestrol* OR hexestrofen* OR hexital* OR hexron* OR "hexypheen" OR "hi bestrol" OR hormoestrol* OR makarol* OR menostilbene* OR "meso hexestrol" OR mesohexestrol* OR micrest* OR microest* OR mikarol* OR milestrol* OR neo estranol* OR "nsc 3070" OR nsc3070* OR "nsc 35752" OR nsc35752* OR "nsc 9894" OR nsc9894* OR oekolp* OR oestramon* OR oestrogeni* OR oestromenin* OR oestromensyl* OR oestrostilben* OR oestrosyntal* OR orestrol* OR oroestron* OR ovendosyn* OR pabestrol* OR pabestrosalve* OR palestrol* OR proestrin* OR serral* OR sinestrol* OR "stil rol" OR stilbarol* OR stilbenol* OR stilbesterol* OR stilbestro* OR stilbetin* OR stilboefral* OR stilboestro* OR stilbol* OR stilkap* OR synestrin* OR syntex* OR synthoestrin* OR synthovo* OR syntrogene* OR tampovagan* OR arolac* OR cuvalit* OR dopergin* OR lisurid* OR lysurid* OR lysenyl* OR "methylergol carbamide" OR nenad* OR prolacam* OR revanil* OR almirid* OR cripar* OR "dhk 135" OR dhk135 OR dihydro alpha ergocryptine* OR dihydroergocriptin* OR dihydroergocryptin* OR dihydroergokriptin* OR dihydroergokryptin* OR myrol* OR vasobral* OR "clavine alkaloid" OR "clavine alkaloids" OR ergot alkaloid* OR "ergot drug" OR "ergot drugs" OR "ergotoxine alkaloid" OR ergoline* OR ergotamine* OR acrofol* OR aerodiol* OR almediol* OR alora* OR altrad* OR aquadiol* OR aquiol* OR bardiol* OR beta estradiol* OR "bio e gel" OR bisteron* OR cerella* OR climaderm* OR climara* OR climera* OR "clinagen la 40" OR compudose* OR "corpa gen" OR corpagen* OR delidose* OR "dep gynogen" OR dermestril* OR dihydro alpha follikelhormon* OR "dihydro oestron" OR dihydrofollicul* OR dihydromenformon* OR dihydrooestron* OR dihydrotheelin* OR dihydroxyestrin* OR dimenformon* OR diogyn* OR divigel* OR "e 2" OR elestrin* OR endofollicolin* OR "ent estradiol" OR esclim* OR estovite* OR estrace* OR estradiol* OR estradot* OR estraguard* OR "estragyn la 5" OR estraldine* OR estran* OR estrasorb* OR estratab* OR estredox* OR estreva* OR estrifam* OR estring* OR estro-cyp* OR estrofem* OR estroform* OR estrogel* OR estrogil* OR estrogyne* OR estrolan OR estrovite* OR evafilm* OR evamist* OR evepia* OR evorel* OR "fem 7" OR fematrix* OR femestral* OR femestrol* OR femiderm* OR femogen OR fempatch* OR femsept* OR femseven* OR femtran* OR folanyl* OR "follicormon erba" OR follicyclin* OR ginedisc* OR gynergon* OR gynodiol* OR gynoestryl* OR gynokadin* OR gynpolar* OR innofem* OR lamdiol* OR lindisc* OR "linoladiol n" OR lipoide* OR "lut ovocycline" OR lyrelle* OR macrodiol* OR "menaval-20" OR meno-mpa* OR "menodin tts" OR menorest* OR menoring* OR menostar* OR mestrace* OR minivelle* OR "nsc 20293" OR nsc20293 OR oesclim* OR oestergon* OR oestradiol* OR oestring* OR oestrodose* OR oestroform* OR oestrogel* OR oestroglandol* OR ormogamma* OR ovahormon* OR ovasterol* OR ovociclin* OR ovocyclin* OR ovocylin* OR perlatanol* OR primofol* OR primogyn* OR profolid* OR profoliol* OR proginon* OR progynon* OR riselle* OR sandrena* OR "sisare gel" OR syndiol* OR systen* OR thais* OR tradelia* OR vagifem* OR valergen* OR vivelle* OR vivelledot* OR zumenon* OR estrogen* OR oestrogen* OR "kober chromogen" OR aethinylstradiol* OR "diogyn e" OR diolyn* OR dyloform* OR "e 4876" OR e4876* OR esteed* OR estigyn* OR estinyl* OR "eston e" OR estoral* OR estrasal* OR estronex OR "estroral pabyrn" OR ethidol* OR ethin oestryl* OR ethinoral* OR ethinyl estradiol* OR ethinyl oestradiol* OR ethinylestradiol* OR ethinyloestradiol* OR "ethynil estradiol" OR ethynilestradiol* OR ethynyl estradiol* OR ethynylestra* OR eticiclin* OR eticyclin* OR eticyclol* OR eticylol* OR etifollin* OR etinestrol* OR etinestryl* OR etinilestradiol* OR etinoestryl* OR etinylestradiol* OR etivex* OR feminone* OR fodinyl* OR follicoral* OR follikoral* OR ginestrene* OR ginormon* OR gynoral* OR hewestrol* OR hewoestrol* OR ibioculine* OR inestra* OR kolpolyn* OR linoral* OR lynestoral* OR lynoral* OR manodiol* OR menolyn* OR metrociclina* OR metroval* OR microfollin* OR mikrofollin* OR nadestryl* OR neo-estrone* OR neo-oestrone* OR neoestrone* OR "norma oestren" OR novestrol* OR "nsc 10973" OR nsc10973* OR nylestin* OR oestralyn* OR oestroperos* OR oradiol* OR orestralyn* OR ostral* OR perovex* OR "primogyn c" OR "primogyn m" OR "progynon c" OR "progynon m" OR methylergobasin* OR methylergobrevin* OR methylergonovin* OR methylergometrin* OR methergin* OR myomergin*).ti,ab,kw,kf |
| 4 | HIV | Exp HIV/ OR exp HIV infections/ OR ((AIDS NOT (decision aids OR hearing aids OR Technological aids OR communication aids)) OR HIV OR acquired immunologic deficiency syndrome* OR acquired immune deficiency syndrome* OR acquired immuno deficiency syndrome* OR acquired immunodeficiency syndrome* OR human immunologic deficiency virus* OR human immune deficiency virus* OR human immuno deficiency virus* OR human immunodeficiency virus* OR immunologic deficiency associated virus* OR immune deficiency associated virus* OR immuno deficiency associated virus* OR immunodeficiency associated virus*).ti,ab,kw,kf |
| 5 | NOT | exp animals/ not exp humans/ |
| 6 | Combinaison | (((1 OR (2 AND 3)) AND 4) NOT 5) |
| 7 | Limitation | Limit 5 to (english or french) = 53 résultats |

## Embase

| 1 | Lactation disorders | Lactation Disorder/ OR ((lactation* OR hypogalactia* OR colostrum* OR milk* OR breastmilk* OR breastfeed* OR breastfed* OR breast feed* OR breast fed*) AND (block* OR inhibit* OR suppress* OR interrupt*)).ti,ab,kw |
| --- | --- | --- |
| 2 | Lactation disorders | Lactation/ OR exp Colostrum/ OR breast milk/ OR exp Breast Feeding/ OR (lactation* OR hypogalactia* OR colostrum* OR milk* OR breastmilk* OR breastfeed* OR breastfed* OR breast feed* OR breast fed*).ti,ab,kw |
| 3 | Inhibition drugs | Bromocriptine/ OR cabergoline/ OR Diethylstilbestrol/ OR Lisuride/ OR Dihydroergocryptine/ OR Ergot Alkaloid/ OR ergoline derivative/ OR ergotamine derivative/ OR Estradiol/ OR Estrogen/ OR ethinylestradiol/ OR methylergometrine/ OR (2 bromo alpha ergokriptin* OR 2 bromo alpha ergokryptin* OR 2 bromo alpha ergocriptin* OR 2 bromo alpha ergocryptin* OR antipark* OR bromed* OR bromergon* OR bromocriptin* OR bromocryptin* OR bromoergocriptin* OR bromoergocryptin* OR bromokin* OR bromopar* OR cb154* OR "cb 154" OR ergobromocriptin* OR ergoset* OR parlodel* OR serocryptin* OR actualene* OR cabarsuss* OR cabaser* OR cabergolin* OR cabest* OR dostinex* OR fce 21336* OR fce21336* OR galastop* OR sogilen* OR sostilar* OR velactis* OR acnestrol* OR agostilben* OR "alpha,alpha' diethyl 4,4' dihydroxybibenzyl" OR "alpha,alpha' diethyl 4,4' stilbenediol" OR apstil* OR boestrol* OR clinestrol* OR clinoestrol* OR comestrol* OR cycloestrol* OR cyren a* OR "d.e.s." OR "di estryl" OR diaethylstilboestrol* OR diethylbestrol* OR diethylstilbestrol* OR diethylstilboestrol* OR dihydrostilbestrol* OR dihydrostilboestrol* OR distilbene* OR domestrol* OR esoestrol* OR estimon* OR estrastilben* OR estrene* OR estrobene* OR estromenin* OR estromonum* OR estrostilben* OR estrosyn* OR ethylstilbestrol* OR ethylstilboestrol* OR folone* OR fonatol* OR grafestrol* OR hexanestrol* OR hexanoestrol* OR hexestrofen* OR hexital* OR hexron* OR "hexypheen" OR "hi bestrol" OR hormoestrol* OR makarol* OR menostilbene* OR "meso hexestrol" OR mesohexestrol* OR micrest* OR microest* OR mikarol* OR milestrol* OR neo estranol* OR "nsc 3070" OR nsc3070* OR "nsc 35752" OR nsc35752* OR "nsc 9894" OR nsc9894* OR oekolp* OR oestramon* OR oestrogeni* OR oestromenin* OR oestromensyl* OR oestrostilben* OR oestrosyntal* OR orestrol* OR oroestron* OR ovendosyn* OR pabestrol* OR pabestrosalve* OR palestrol* OR proestrin* OR serral* OR sinestrol* OR "stil rol" OR stilbarol* OR stilbenol* OR stilbesterol* OR stilbestro* OR stilbetin* OR stilboefral* OR stilboestro* OR stilbol* OR stilkap* OR synestrin* OR syntex* OR synthoestrin* OR synthovo* OR syntrogene* OR tampovagan* OR arolac* OR cuvalit* OR dopergin* OR lisurid* OR lysurid* OR lysenyl* OR "methylergol carbamide" OR nenad* OR prolacam* OR revanil* OR almirid* OR cripar* OR "dhk 135" OR dhk135 OR dihydro alpha ergocryptine* OR dihydroergocriptin* OR dihydroergocryptin* OR dihydroergokriptin* OR dihydroergokryptin* OR myrol* OR vasobral* OR "clavine alkaloid" OR "clavine alkaloids" OR ergot alkaloid* OR "ergot drug" OR "ergot drugs" OR "ergotoxine alkaloid" OR ergoline* OR ergotamine* OR acrofol* OR aerodiol* OR almediol* OR alora* OR altrad* OR aquadiol* OR aquiol* OR bardiol* OR beta estradiol* OR "bio e gel" OR bisteron* OR cerella* OR climaderm* OR climara* OR climera* OR "clinagen la 40" OR compudose* OR "corpa gen" OR corpagen* OR delidose* OR "dep gynogen" OR dermestril* OR dihydro alpha follikelhormon* OR "dihydro oestron" OR dihydrofollicul* OR dihydromenformon* OR dihydrooestron* OR dihydrotheelin* OR dihydroxyestrin* OR dimenformon* OR diogyn* OR divigel* OR "e 2" OR elestrin* OR endofollicolin* OR "ent estradiol" OR esclim* OR estovite* OR estrace* OR estradiol* OR estradot* OR estraguard* OR "estragyn la 5" OR estraldine* OR estran* OR estrasorb* OR estratab* OR estredox* OR estreva* OR estrifam* OR estring* OR estro-cyp* OR estrofem* OR estroform* OR estrogel* OR estrogil* OR estrogyne* OR estrolan OR estrovite* OR evafilm* OR evamist* OR evepia* OR evorel* OR "fem 7" OR fematrix* OR femestral* OR femestrol* OR femiderm* OR femogen OR fempatch* OR femsept* OR femseven* OR femtran* OR folanyl* OR "follicormon erba" OR follicyclin* OR ginedisc* OR gynergon* OR gynodiol* OR gynoestryl* OR gynokadin* OR gynpolar* OR innofem* OR lamdiol* OR lindisc* OR "linoladiol n" OR lipoide* OR "lut ovocycline" OR lyrelle* OR macrodiol* OR "menaval-20" OR meno-mpa* OR "menodin tts" OR menorest* OR menoring* OR menostar* OR mestrace* OR minivelle* OR "nsc 20293" OR nsc20293 OR oesclim* OR oestergon* OR oestradiol* OR oestring* OR oestrodose* OR oestroform* OR oestrogel* OR oestroglandol* OR ormogamma* OR ovahormon* OR ovasterol* OR ovociclin* OR ovocyclin* OR ovocylin* OR perlatanol* OR primofol* OR primogyn* OR profolid* OR profoliol* OR proginon* OR progynon* OR riselle* OR sandrena* OR "sisare gel" OR syndiol* OR systen* OR thais* OR tradelia* OR vagifem* OR valergen* OR vivelle* OR vivelledot* OR zumenon* OR estrogen* OR oestrogen* OR "kober chromogen" OR aethinylstradiol* OR "diogyn e" OR diolyn* OR dyloform* OR "e 4876" OR e4876* OR esteed* OR estigyn* OR estinyl* OR "eston e" OR estoral* OR estrasal* OR estronex OR "estroral pabyrn" OR ethidol* OR ethin oestryl* OR ethinoral* OR ethinyl estradiol* OR ethinyl oestradiol* OR ethinylestradiol* OR ethinyloestradiol* OR "ethynil estradiol" OR ethynilestradiol* OR ethynyl estradiol* OR ethynylestra* OR eticiclin* OR eticyclin* OR eticyclol* OR eticylol* OR etifollin* OR etinestrol* OR etinestryl* OR etinilestradiol* OR etinoestryl* OR etinylestradiol* OR etivex* OR feminone* OR fodinyl* OR follicoral* OR follikoral* OR ginestrene* OR ginormon* OR gynoral* OR hewestrol* OR hewoestrol* OR ibioculine* OR inestra* OR kolpolyn* OR linoral* OR lynestoral* OR lynoral* OR manodiol* OR menolyn* OR metrociclina* OR metroval* OR microfollin* OR mikrofollin* OR nadestryl* OR neo-estrone* OR neo-oestrone* OR neoestrone* OR "norma oestren" OR novestrol* OR "nsc 10973" OR nsc10973* OR nylestin* OR oestralyn* OR oestroperos* OR oradiol* OR orestralyn* OR ostral* OR perovex* OR "primogyn c" OR "primogyn m" OR "progynon c" OR "progynon m" OR methylergobasin* OR methylergobrevin* OR methylergonovin* OR methylergometrin* OR methergin* OR myomergin*).ti,ab,kw |
| 4 | HIV | Exp Human immunodeficiency virus/ OR exp Human immunodeficiency virus infection/ OR ((AIDS NOT (decision aids OR hearing aids OR Technological aids OR communication aids)) OR HIV OR acquired immunologic deficiency syndrome* OR acquired immune deficiency syndrome* OR acquired immuno deficiency syndrome* OR acquired immunodeficiency syndrome* OR human immunologic deficiency virus* OR human immune deficiency virus* OR human immuno deficiency virus* OR human immunodeficiency virus* OR immunologic deficiency associated virus* OR immune deficiency associated virus* OR immuno deficiency associated virus* OR immunodeficiency associated virus*).ti,ab,kw |
| 5 | NOT | exp animal/ not exp human/ |
| 6 | Combinaison | (((1 OR (2 AND 3)) AND 4) NOT 5) |
| 7 | Limitation | Limit 5 to (english or french) = 423 résultats |

## Web of Science

| 1 | Lactation disorders | TS=((lactation* OR hypogalactia* OR colostrum* OR milk* OR breastmilk* OR breastfeed* OR breastfed* OR breast feed* OR breast fed*) AND (block* OR inhibit* OR suppress* OR interrupt*)) |
| --- | --- | --- |
| 2 | Lactation | TS=(lactation* OR hypogalactia* OR colostrum* OR milk* OR breastmilk* OR breastfeed* OR breastfed* OR breast feed* OR breast fed*) |
| 3 | Inhibition drug | TS=("bromo alpha ergokriptin" OR "bromo alpha ergokryptin" OR "bromo alpha ergocriptin" OR "bromo alpha ergocryptin" OR antipark* OR bromed* OR bromergon* OR bromocriptin* OR bromocryptin* OR bromoergocriptin* OR bromoergocryptin* OR bromokin* OR bromopar* OR cb154* OR "cb 154" OR ergobromocriptin* OR ergoset* OR parlodel* OR serocryptin* OR actualene* OR cabarsuss* OR cabaser* OR cabergolin* OR cabest* OR dostinex* OR fce 21336* OR fce21336* OR galastop* OR sogilen* OR sostilar* OR velactis* OR acnestrol* OR agostilben* OR "alpha,alpha' diethyl 4,4' dihydroxybibenzyl" OR "alpha,alpha' diethyl 4,4' stilbenediol" OR apstil* OR boestrol* OR clinestrol* OR clinoestrol* OR comestrol* OR cycloestrol* OR cyren a OR "d.e.s." OR "di estryl" OR diaethylstilboestrol* OR diethylbestrol* OR diethylstilbestrol* OR diethylstilboestrol* OR dihydrostilbestrol* OR dihydrostilboestrol* OR distilbene* OR domestrol* OR esoestrol* OR estimon* OR estrastilben* OR estrene* OR estrobene* OR estromenin* OR estromonum* OR estrostilben* OR estrosyn* OR ethylstilbestrol* OR ethylstilboestrol* OR folone* OR fonatol* OR grafestrol* OR hexanestrol* OR hexanoestrol* OR hexestrofen* OR hexital* OR hexron* OR "hexypheen" OR "hi bestrol" OR hormoestrol* OR makarol* OR menostilbene* OR "meso hexestrol" OR mesohexestrol* OR micrest* OR microest* OR mikarol* OR milestrol* OR neo estranol* OR "nsc 3070" OR nsc3070* OR "nsc 35752" OR nsc35752* OR "nsc 9894" OR nsc9894* OR oekolp* OR oestramon* OR oestrogeni* OR oestromenin* OR oestromensyl* OR oestrostilben* OR oestrosyntal* OR orestrol* OR oroestron* OR ovendosyn* OR pabestrol* OR pabestrosalve* OR palestrol* OR proestrin* OR serral* OR sinestrol* OR "stil rol" OR stilbarol* OR stilbenol* OR stilbesterol* OR stilbestro* OR stilbetin* OR stilboefral* OR stilboestro* OR stilbol* OR stilkap* OR synestrin* OR syntex* OR synthoestrin* OR synthovo* OR syntrogene* OR tampovagan* OR arolac* OR cuvalit* OR dopergin* OR lisurid* OR lysurid* OR lysenyl* OR "methylergol carbamide" OR nenad* OR prolacam* OR revanil* OR almirid* OR cripar* OR "dhk 135" OR dhk135 OR dihydro alpha ergocryptine* OR dihydroergocriptin* OR dihydroergocryptin* OR dihydroergokriptin* OR dihydroergokryptin* OR myrol* OR vasobral* OR "clavine alkaloid" OR "clavine alkaloids" OR ergot alkaloid* OR "ergot drug" OR "ergot drugs" OR "ergotoxine alkaloid" OR ergoline* OR ergotamine* OR acrofol* OR aerodiol* OR almediol* OR alora* OR altrad* OR aquadiol* OR aquiol* OR bardiol* OR beta estradiol* OR "bio e gel" OR bisteron* OR cerella* OR climaderm* OR climara* OR climera* OR "clinagen la 40" OR compudose* OR "corpa gen" OR corpagen* OR delidose* OR "dep gynogen" OR dermestril* OR dihydro alpha follikelhormon* OR "dihydro oestron" OR dihydrofollicul* OR dihydromenformon* OR dihydrooestron* OR dihydrotheelin* OR dihydroxyestrin* OR dimenformon* OR diogyn* OR divigel* OR "e 2" OR elestrin* OR endofollicolin* OR "ent estradiol" OR esclim* OR estovite* OR estrace* OR estradiol* OR estradot* OR estraguard* OR "estragyn la 5" OR estraldine* OR estran* OR estrasorb* OR estratab* OR estredox* OR estreva* OR estrifam* OR estring* OR estro-cyp* OR estrofem* OR estroform* OR estrogel* OR estrogil* OR estrogyne* OR estrolan OR estrovite* OR evafilm* OR evamist* OR evepia* OR evorel* OR "fem 7" OR fematrix* OR femestral* OR femestrol* OR femiderm* OR femogen OR fempatch* OR femsept* OR femseven* OR femtran* OR folanyl* OR "follicormon erba" OR follicyclin* OR ginedisc* OR gynergon* OR gynodiol* OR gynoestryl* OR gynokadin* OR gynpolar* OR innofem* OR lamdiol* OR lindisc* OR "linoladiol n" OR lipoide* OR "lut ovocycline" OR lyrelle* OR macrodiol* OR "menaval-20" OR meno-mpa* OR "menodin tts" OR menorest* OR menoring* OR menostar* OR mestrace* OR minivelle* OR "nsc 20293" OR nsc20293 OR oesclim* OR oestergon* OR oestradiol* OR oestring* OR oestrodose* OR oestroform* OR oestrogel* OR oestroglandol* OR ormogamma* OR ovahormon* OR ovasterol* OR ovociclin* OR ovocyclin* OR ovocylin* OR perlatanol* OR primofol* OR primogyn* OR profolid* OR profoliol* OR proginon* OR progynon* OR riselle* OR sandrena* OR "sisare gel" OR syndiol* OR systen* OR thais* OR tradelia* OR vagifem* OR valergen* OR vivelle* OR vivelledot* OR zumenon* OR estrogen* OR oestrogen* OR "kober chromogen" OR aethinylstradiol* OR "diogyn e" OR diolyn* OR dyloform* OR "e 4876" OR e4876* OR esteed* OR estigyn* OR estinyl* OR "eston e" OR estoral* OR estrasal* OR estronex OR "estroral pabyrn" OR ethidol* OR ethin oestryl* OR ethinoral* OR ethinyl estradiol* OR ethinyl oestradiol* OR ethinylestradiol* OR ethinyloestradiol* OR "ethynil estradiol" OR ethynilestradiol* OR ethynyl estradiol* OR ethynylestra* OR eticiclin* OR eticyclin* OR eticyclol* OR eticylol* OR etifollin* OR etinestrol* OR etinestryl* OR etinilestradiol* OR etinoestryl* OR etinylestradiol* OR etivex* OR feminone* OR fodinyl* OR follicoral* OR follikoral* OR ginestrene* OR ginormon* OR gynoral* OR hewestrol* OR hewoestrol* OR ibioculine* OR inestra* OR kolpolyn* OR linoral* OR lynestoral* OR lynoral* OR manodiol* OR menolyn* OR metrociclina* OR metroval* OR microfollin* OR mikrofollin* OR nadestryl* OR neo-estrone* OR neo-oestrone* OR neoestrone* OR "norma oestren" OR novestrol* OR "nsc 10973" OR nsc10973* OR nylestin* OR oestralyn* OR oestroperos* OR oradiol* OR orestralyn* OR ostral* OR perovex* OR "primogyn c" OR "primogyn m" OR "progynon c" OR "progynon m" OR methylergobasin* OR methylergobrevin* OR methylergonovin* OR methylergometrin* OR methergin* OR myomergin*) |
| 4 | HIV | TS=(("AIDS" NOT (decision aids OR hearing aids OR Technological aids OR communication aids)) OR HIV OR acquired immunologic deficiency syndrome* OR acquired immune deficiency syndrome* OR acquired immuno deficiency syndrome* OR acquired immunodeficiency syndrome* OR human immunologic deficiency virus* OR human immune deficiency virus* OR human immuno deficiency virus* OR human immunodeficiency virus* OR immunologic deficiency associated virus* OR immune deficiency associated virus* OR immuno deficiency associated virus* OR immunodeficiency associated virus*) |
| 5 | Combinaison | ((#1 OR (#2 AND #3)) AND #4) AND LANGUAGE: (English OR French) = 474 résultats |

## Scopus

( ( ( TITLE-ABS-KEY ( lactation* OR hypogalactia* OR colostrum* OR milk* OR breastmilk* OR breastfeed* OR breastfed* OR "breast feed" OR "breast feeding" OR "breast feeds" OR "breast fed" ) ) AND ( TITLE-ABS-KEY ( "bromo alpha ergokriptin" OR "bromo alpha ergokryptin" OR "bromo alpha ergocriptin" OR "bromo alpha ergocryptin" OR antipark* OR bromed* OR bromergon* OR bromocriptin* OR bromocryptin* OR bromoergocriptin* OR bromoergocryptin* OR bromokin* OR bromopar* OR cb154* OR "cb 154" OR ergobromocriptin* OR ergoset* OR parlodel* OR serocryptin* OR actualene* OR cabarsuss* OR cabaser* OR cabergolin* OR cabest* OR dostinex* OR "fce 21336" OR fce21336 OR galastop* OR sogilen* OR sostilar* OR velactis* OR acnestrol* OR agostilben* OR "alpha,alpha' diethyl 4,4' dihydroxybibenzyl" OR "alpha,alpha' diethyl 4,4' stilbenediol" OR apstil* OR boestrol* OR clinestrol* OR clinoestrol* OR comestrol* OR cycloestrol* OR "cyren a" OR "d.e.s." OR "di estryl" OR diaethylstilboestrol* OR diethylbestrol* OR diethylstilbestrol* OR diethylstilboestrol* OR dihydrostilbestrol* OR dihydrostilboestrol* OR distilbene* OR domestrol* OR esoestrol* OR estimon* OR estrastilben* OR estrene* OR estrobene* OR estromenin* OR estromonum* OR estrostilben* OR estrosyn* OR ethylstilbestrol* OR ethylstilboestrol* OR folone* OR fonatol* OR grafestrol* OR hexanestrol* OR hexanoestrol* OR hexestrofen* OR hexital* OR hexron* OR "hexypheen" OR "hi bestrol" OR hormoestrol* OR makarol* OR menostilbene* OR "meso hexestrol" OR mesohexestrol* OR micrest* OR microest* OR mikarol* OR milestrol* OR "neo estranol" OR "nsc 3070" OR nsc3070 OR "nsc 35752" OR nsc35752 OR "nsc 9894" OR nsc9894* OR oekolp* OR oestramon* OR oestrogeni* OR oestromenin* OR oestromensyl* OR oestrostilben* OR oestrosyntal* OR orestrol* OR oroestron* OR ovendosyn* OR pabestrol* OR pabestrosalve* OR palestrol* OR proestrin* OR serral* OR sinestrol* OR "stil rol" OR stilbarol* OR stilbenol* OR stilbesterol* OR stilbestro* OR stilbetin* OR stilboefral* OR stilboestro* OR stilbol* OR stilkap* OR synestrin* OR syntex* OR synthoestrin* OR synthovo* OR syntrogene* OR tampovagan* OR arolac* OR cuvalit* OR dopergin* OR lisurid* OR lysurid* OR lysenyl* OR "methylergol carbamide" OR nenad* OR prolacam* OR revanil* OR almirid* OR cripar* OR "dhk 135" OR dhk135 OR "dihydro alpha ergocryptine" OR dihydroergocriptin* OR dihydroergocryptin* OR dihydroergokriptin* OR dihydroergokryptin* OR myrol* OR vasobral* OR "clavine alkaloid" OR "clavine alkaloids" OR "ergot alkaloid" OR "ergot alkaloids" OR "ergot drug" OR "ergot drugs" OR "ergotoxine alkaloid" OR ergoline* OR ergotamine* OR acrofol* OR aerodiol* OR almediol* OR alora* OR altrad* OR aquadiol* OR aquiol* OR bardiol* OR "beta estradiol" OR "bio e gel" OR bisteron* OR cerella* OR climaderm* OR climara* OR climera* OR "clinagen la 40" OR compudose* OR "corpa gen" OR corpagen* OR delidose* OR "dep gynogen" OR dermestril* OR "dihydro alpha follikelhormon" OR "dihydro oestron" OR dihydrofollicul* OR dihydromenformon* OR dihydrooestron* OR dihydrotheelin* OR dihydroxyestrin* OR dimenformon* OR diogyn* OR divigel* OR elestrin* OR endofollicolin* OR "ent estradiol" OR esclim* OR estovite* OR estrace* OR estradiol* OR estradot* OR estraguard* OR "estragyn la 5" OR estraldine* OR estran* OR estrasorb* OR estratab* OR estredox* OR estreva* OR estrifam* OR estring* OR estro-cyp* OR estrofem* OR estroform* OR estrogel* OR estrogil* OR estrogyne* OR estrolan OR estrovite* OR evafilm* OR evamist* OR evepia* OR evorel* OR "fem 7" OR fematrix* OR femestral* OR femestrol* OR femiderm* OR femogen OR fempatch* OR femsept* OR femseven* OR femtran* OR folanyl* OR "follicormon erba" OR follicyclin* OR ginedisc* OR gynergon* OR gynodiol* OR gynoestryl* OR gynokadin* OR gynpolar* OR innofem* OR lamdiol* OR lindisc* OR "linoladiol n" OR lipoide* OR "lut ovocycline" OR lyrelle* OR macrodiol* OR "menaval-20" OR "meno-mpa" OR "menodin tts" OR menorest* OR menoring* OR menostar* OR mestrace* OR minivelle* OR "nsc 20293" OR nsc20293 OR oesclim* OR oestergon* OR oestradiol* OR oestring* OR oestrodose* OR oestroform* OR oestrogel* OR oestroglandol* OR ormogamma* OR ovahormon* OR ovasterol* OR ovociclin* OR ovocyclin* OR ovocylin* OR perlatanol* OR primofol* OR primogyn* OR profolid* OR profoliol* OR proginon* OR progynon* OR riselle* OR sandrena* OR "sisare gel" OR syndiol* OR systen* OR thais* OR tradelia* OR vagifem* OR valergen* OR vivelle* OR vivelledot* OR zumenon* OR estrogen* OR oestrogen* OR "kober chromogen" OR aethinylstradiol* OR "diogyn e" OR diolyn* OR dyloform* OR "e 4876" OR e4876* OR esteed* OR estigyn* OR estinyl* OR "eston e" OR estoral* OR estrasal* OR estronex OR "estroral pabyrn" OR ethidol* OR "ethin oestryl" OR ethinoral* OR "ethinyl estradiol" OR "ethinyl oestradiol" OR ethinylestradiol* OR ethinyloestradiol* OR "ethynil estradiol" OR ethynilestradiol* OR "ethynyl estradiol" OR ethynylestra* OR eticiclin* OR eticyclin* OR eticyclol* OR eticylol* OR etifollin* OR etinestrol* OR etinestryl* OR etinilestradiol* OR etinoestryl* OR etinylestradiol* OR etivex* OR feminone* OR fodinyl* OR follicoral* OR follikoral* OR ginestrene* OR ginormon* OR gynoral* OR hewestrol* OR hewoestrol* OR ibioculine* OR inestra* OR kolpolyn* OR linoral* OR lynestoral* OR lynoral* OR manodiol* OR menolyn* OR metrociclina* OR metroval* OR microfollin* OR mikrofollin* OR nadestryl* OR "neo-estrone" OR "neo-oestrone" OR neoestrone* OR "norma oestren" OR novestrol* OR "nsc 10973" OR nsc10973 OR nylestin* OR oestralyn* OR oestroperos* OR oradiol* OR orestralyn* OR ostral* OR perovex* OR "primogyn c" OR "primogyn m" OR "progynon c" OR "progynon m" OR methylergobasin* OR methylergobrevin* OR methylergonovin* OR methylergometrin* OR methergin* OR myomergin* ) ) ) OR ( TITLE-ABS-KEY ( ( lactation* OR hypogalactia* OR colostrum* OR milk* OR breastmilk* OR breastfeed* OR breastfed* OR "breast feed" OR "breast feeding" OR "breast feeds" OR "breast fed" ) AND ( block* OR inhibit* OR suppress* OR interrupt* ) ) ) ) AND ( ( TITLE-ABS-KEY ( ( ( hiv OR "acquired immunologic deficiency syndrome" OR "acquired immunologic deficiency syndromeS" OR "acquired immune deficiency syndrome" OR "acquired immune deficiency syndromeS" OR "acquired immuno deficiency syndrome" OR "acquired immuno deficiency syndromes" OR "acquired immunodeficiency syndrome" OR "acquired immunodeficiency syndromes" OR "human immunologic deficiency virus" OR "human immunologic deficiency viruses" OR "human immune deficiency virus" OR "human immune deficiency viruses" OR "human immuno deficiency virus" OR "human immuno deficiency viruses" ) ) ) ) OR ( ( TITLE-ABS-KEY ( ( ( "AIDS" ) ) ) ) AND NOT ( TITLE-ABS-KEY ( ( ( "decision aids" OR "hearing aids" OR "Technological aids" OR "communication aids" ) ) ) ) ) ) AND ( LIMIT-TO ( LANGUAGE , "English" ) OR LIMIT-TO ( LANGUAGE , "French" ) ) = 725 résultats
